# Indication of 3_10_-Helix Structure in Gas-Phase Neutral Pentaalanine

**DOI:** 10.1021/acs.jpca.2c07863

**Published:** 2023-01-20

**Authors:** Åke Andersson, Vasyl Yatsyna, Mathieu Linares, Anouk Rijs, Vitali Zhaunerchyk

**Affiliations:** † Department of Physics, University of Gothenburg, 41296 Gothenburg, Sweden; ‡ FELIX Laboratory, Institute for Molecules and Materials, Radboud University, Toernooiveld 7, 6525 ED Nijmegen, The Netherlands; § Laboratoire de Chimie Physique Moléculaire, École Polytechnique Fédérale de Lausanne, EPFL SB ISIC LCPM, Station 6, CH-1015 Lausanne, Switzerland; ∥ Laboratory of Organic Electronics and Group of Scientific Visualization Department of Science and Technology (ITN), Linköping University, 601 74 Norrköping, Sweden; ⊥ Division of BioAnalytical Chemistry, AIMMS Amsterdam Institute of Molecular and Life Sciences, Vrije Universiteit Amsterdam, De Boelelaan 1108, 1081 HV, Amsterdam, The Netherlands

## Abstract

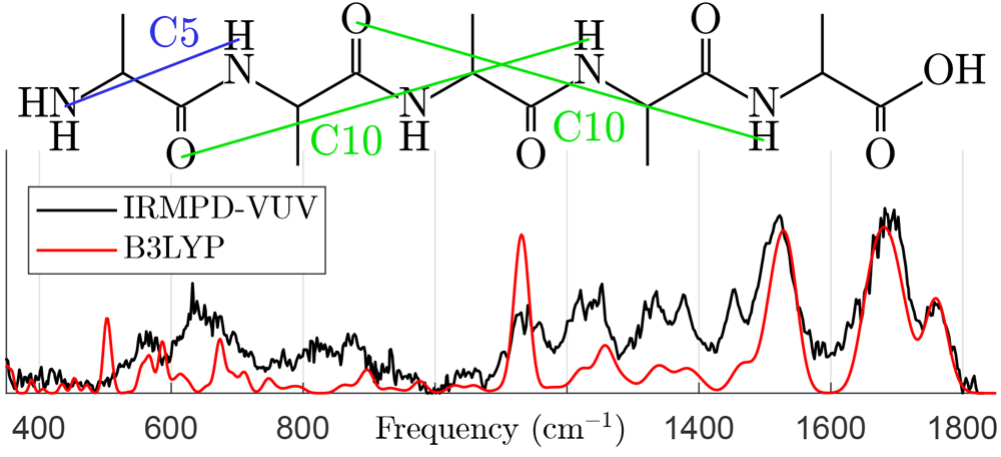

We investigate the gas-phase structure of the neutral pentaalanine peptide. The IR spectrum in the 340–1820 cm^–1^ frequency range is obtained by employing supersonic jet cooling, infrared multiphoton dissociation, and vacuum-ultraviolet action spectroscopy. Comparison with quantum chemical spectral calculations suggests that the molecule assumes multiple stable conformations, mainly of two structure types. In the most stable conformation theoretically found, the N-terminus forms a C5 ring and the backbone resembles that of an 3_10_-helix with two β-turns. Additionally, the conformational preferences of pentaalanine have been evaluated using Born–Oppenheimer molecular dynamics, showing that a nonzero simulation time step causes a systematic frequency shift.

## 1 Introduction

Peptides are small polymers of amino acids and ideal for the study of local interactions in proteins. Their relatively small size allows their delivery into the gas phase, where infrared (IR) spectroscopy can be applied to the isolated molecules. The IR absorption frequencies correspond to vibrational modes and are sensitive enough to act as a conformer-specific fingerprint.^1,2^ When combined with theoretical predictions, IR spectroscopy can be used to deduce the molecular structure of these peptides and other small- to medium-sized biomolecules.

In gas-phase studies where the sample density is low, direct absorption spectroscopy suffers from a low signal-to-background ratio. Therefore, the preferred method is action spectroscopy, where the signal comes from a change in some property of the sample molecule upon absorption. One instance is IR multiple-photon dissociation (IRMPD), where the combination of the absorption of multiple photons and intramolecular vibrational relaxation results in fragmentation. By measuring the fragmentation yield as a function of the IR wavelength, an IR action spectrum can be recorded. IRMPD combined with an ion cyclotron trap has been widely applied to study gas-phase ions.^3−8^ Single-photon spectra can be obtained with the messenger-tagging method. The trapped ions are made to form weakly bound complexes with a gas adduct such as N_2_. Single-photon absorption then leads to detachment of the adduct, and the change in mass is used as a signal.^9−11^

For neutral molecules, there are action spectroscopy techniques involving ionization. In infrared–ultraviolet (IR–UV) ion-dip spectroscopy, two UV photons resonantly ionize a specific conformation of the molecule, in competition with IR absorption.^12,13^ That is, IR photon absorption hampers resonant UV ionization. For the resonant ionization to occur, the molecule must have a UV chromophore, most commonly an aromatic ring. We have previously demonstrated that IRMPD when combined with a vacuum-ultraviolet (VUV) single-photon ionization can be applied also to neutral molecular beams, without the need for such a UV chromophore.^14^ This is valuable because 17 out of the 20 natural amino acids lack a chromophore.

Polyalanine peptides in particular are interesting because they are minimal model systems for secondary structures. For instance, they form α-helices in physiological solution^15,16^ and are predicted to adopt a helical structure in the gas phase.^17^ Until recently, it has not been possible to perform gas-phase IRMPD experiments on neutral peptides, and thus studies have been limited to charged species. Examples include not only protonated polyalanines Ala_{2–5,7}_H^+^^18,19^ but also metal cation polypeptide complexes such as Ala_*n*_X (X = Na^+^, Ka^+^, Ca^2+^, etc*.*).^20,21^

Neutral peptides similar to polyalanines have also been studied. The capped peptides Ac-Ala-Phe-Ala-NH_2_^22^ and Ac-Aib-Phe-Aib-NH_2_^23^ have been investigated with IR–UV ion-dip spectroscopy and have been found to have a structure similar to that of a 3_10_-helix. These molecules were in part stabilized by the phenyl group, so it is interesting to see if the structure is similar for a pure alanine peptide.

In this work, we apply the IRMPD–VUV method to study neutral pentaalanine (Ala_5_) having natural chirality. The purpose is twofold: First, we aim to compare the IRMPD spectrum of Ala_5_ with the predicted spectra of likely abundant conformers. Second, with this experiment we challenge the IRMPD–VUV method by studying a large, floppy molecule. We have previously successfully applied the method on diglycine^24^ and dialanine,^25^ but to our knowledge larger chromophore-free neutral peptides have not been investigated with IR action spectroscopy.

On the theoretical side, in addition to harmonic frequency analysis, we use the alternative dynamical spectrum calculation method pioneered by Gaigeot.^26,27^ This molecular dynamics-based method has the advantage of not making either of the two harmonic approximations (force and polarization responses), which one hopes would lead to better treatment of anharmonic effects. Indeed, the method has been applied with great success^28^ in the far-IR range, where the vibrational modes are generally more anharmonically coupled.

## 2 Methods

### 2.1 Spectroscopic Experiment and Model

The experiment was performed at the FELIX (Free-Electron Lasers for Infrared eXperiments) Laboratory, specifically at the laser desorption molecular beam setup.^1^ We have previously described how IRMPD–VUV spectroscopy is performed with this setup,^24^ but we will recapitulate. Ala_5_ molecules were delivered into the gas phase using laser desorption and then seeded into a supersonic jet of argon gas. The supersonic jet expansion cooled down most of the molecules to their rovibrational ground state. The central part of the expanded jet was selected using a skimmer and passed into the interaction chamber, where it intersects two laser pulses. The first pulse was from FELIX and served to dissociate molecules via the IRMPD process. Its frequency was tuned in the range of 340–1820 cm^–1^, its pulse duration was approximately 7 μs, and its power was 30–80 mW depending on the IR frequency. The second pulse was from a Xe–Ar gas cell pumped with the third harmonic of a Nd:YAG laser and served to ionize Ala_5_ and its fragments. It had a wavelength of 118 nm, pulse duration of 3 ns, and estimated pulse energy of 1 μJ. Finally, the produced ions were mass analyzed and detected using a reflectron-type time-of-flight (TOF) mass spectrometer.

An idealized model, used to infer the IR cross-section σ(ν) from the measured ion counts, is as follows: Ala_5_ molecules enter the interaction region neutral and in the ground state. During exposure to the FELIX beam with frequency ν and photon flux Φ(ν), each molecule every short time d*t* has a chance Φ*σ* d*t* to become vibrationally excited. If this happens, then the cross-section for absorption of further IR photons increases drastically and the molecule eventually dissociates. When the exposure ends after τ time, the ratio of intact molecules is now exp(−Φ*στ*). The second VUV pulse then ionizes a fraction of the Ala_5_ and its fragments, and we assume that the ionization probability of the parent Ala_5_ is equal to the sum of the probabilities of its fragments. During or after ionization, some unknown ratio of Ala_5_^+^ fragments into fragments, one of which is an ion. The relative intensities of produced ions are then detected with efficiency independent of mass.

To compensate for the possibility that some unknown ratio of Ala_5_^+^ dissociates during or after ionization, we measure the ion signal with IR laser light both on and off. Specifically, the FELIX IR laser is operated at a repetition rate of 5 Hz while the Nd:YAG runs at 10 Hz, meaning that every second ionization pulse probes molecules without IR irradiation. From the model above, we assert that the IR cross-section can be calculated as
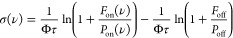
1where *P* is the Ala_5_^+^ count, *F* is the sum of fragment ion counts, and the subscript tells whether IR laser light is on or off. The photon fluence Φτ is proportional to the number of photons in a FELIX macropulse. The second term on the right-hand side is necessary to compensate for the fact that some Ala_5_ dissociates during or after ionization. Because it does not involve the IR laser beam, it is independent of ν.

### 2.2 Quantum-Chemical Calculations

The conformational space of Ala_5_ was analyzed using Tinker.^29^ Specifically, the basin-hopping scan program was used to find 1139 local minima, which were then optimized with the MM3 force field. The 50 lowest energy conformers were further optimized at the DFT level in Gaussian,^30^ using the B3LYP functional with GD3BJ empirical dispersion and the Jun-cc-pVTZ basis set. This basis set is a subset of Aug-cc-pVTZ with similar performance.^31^ Harmonic frequency analyses were performed at the same level. Finally, single-point CBS-4 M calculations were employed to obtain Gibbs energies at 400 K, an estimate of the temperature during laser desorption.^24,25,32,33^ The accuracy of the CBS-4 M method is estimated to be 2 kcal/mol based on benchmarks to the G2 test set.^34,35^

As a measure of conformer distance, we used the minimal (across Euclidean transformations and atom reindexing) root sum square of atom displacements (MRSSAD), which can be swiftly computed.^36^ It has the intuitive properties of being positive for distinct conformers and obeying the triangle inequality. While it is possible to weight the summed atomwise contributions, all applications in this work use equal weight for every atom.

We used the harmonic frequency analyses as chief predictions of spectra, with two minor adjustments to each: First, the frequencies were multiplied by a scaling factor of 0.98, which is an empirical constant specific to B3LYP.^37,38^ Second, the spectrum was broadened by converting each calculated IR band with frequency ν and intensity *I* to a Gaussian function with mean ν and integrated value *I*. The width σ was taken to match the spectral line width of FELIX, estimated to be σ_FELIX_ = 0.01ν.

We also employ Born–Oppenheimer molecular dynamics (BOMD) for dynamical IR spectra prediction. In a BOMD simulation, the nuclei are classical point masses affected by Hellmann–Feynman forces. We simulated BOMD of Ala_5_ at the B3LYP/N07D level for a duration of 2 to 3 ps. The IR absorbance cross-section was then estimated from the power spectral density of the electric dipole^39^ and averaged over 30 runs. The next subsection gives a full description of the simulation.

The NCI method^40^ was used to evaluate the strength of intramolecular interactions and assign hydrogen bonds (H-bonds). For every point in a real-space grid covering the molecule, the electron density ρ and its first two derivatives were evaluated. That point was then considered to be in an H-bond if the following three conditions were met:
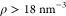
2
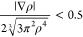
3
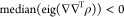
4The threshold value of 18 nm^–3^ is somewhat arbitrary but should not be much higher; otherwise, C5 and C7 ring structures would not be recognized. An evaluation of the left-hand sides in eqs 2–4 was carried out in Multiwfn.^41^

### 2.3 Molecular Dynamics Spectrum

As an alternative to harmonic frequency analysis, the IR spectrum of a molecule can be calculated from BOMD trajectories. This method is naturally divided into two tasks: running BOMD simulations and processing the resulting trajectories.

BOMD simulations were carried out in Gaussian 16^30^ using the ADMP keyword^42−44^ with the FullSCF option at the B3LYP/N07D level. Each simulation had a time range of τ = 2 to 3 ps and a time step of 0.5 fs. The initial positions were taken from equilibrium geometry, and the initial velocities were taken from a Maxwell–Boltzmann distribution with a temperature parameter twice that of the simulation temperature *T*. Thus, each vibrational mode had an ensemble average energy of *k*_B_*T*. Linear and angular momenta were removed by means of projection.

The absorbance cross-section is estimated from the time-dependent electric dipole moment μ(*t*) obtained from simulations. Specifically, the Fermi golden rule is used to relate the absorbance to the Fourier transform of the autocorrelation function of the dipole moment.^26^ When the Wiener–Khintchine theorem is applied, the latter can be simplified to the power spectral density of the dipole moment:
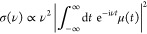
5In practice, the Fourier transform is discrete, padded, and preceded by multiplication with a Hann window function in order to reduce spectral leakage.^45^ The spectrum obtained in this way depends on the random initial velocities and is therefore averaged over 30 BOMD simulations.

The dynamic spectra are adjusted similarly to the harmonic spectra by scaling frequencies with 0.98. They are also broadened in a different way because the dynamic spectra are already broadened in a sense. When applying the padded Fourier transform, the frequency resolution is limited by the finite trajectory length τ. This can be thought of as a broadening with a standard deviation of . Therefore, the second broadening is done with a Gaussian function with variance  to make up the difference.

## 3 Results and Discussion

### 3.1 Ala_5_ Conformers

The conformational search and Gibbs energy calculations found that the top 8 (20) conformers account for 95% (99.9%) of the abundance, assuming Boltzmann populations. Therefore, we will focus on the 8 most stable conformers. Figure 2 shows data of interest for these.

**Figure 1:**
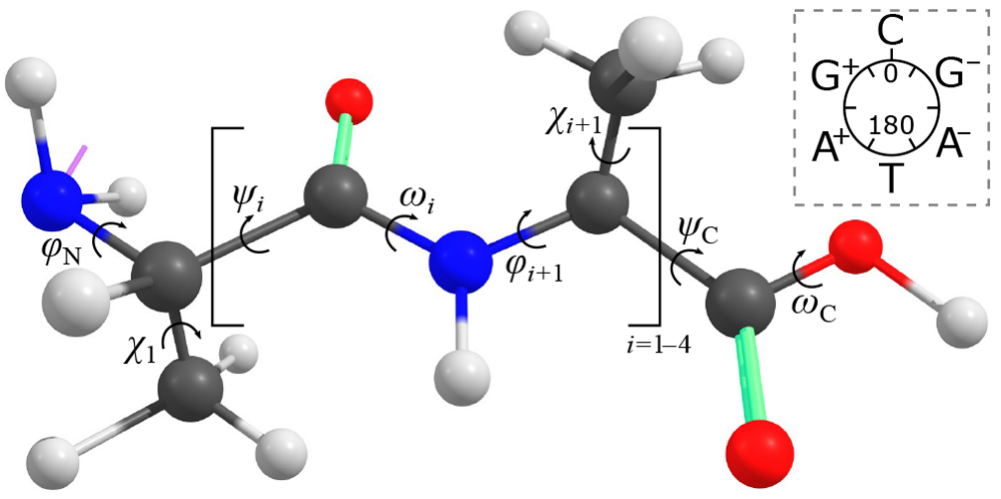
Torsional angles in Ala_5_ exemplified in a fictional conformation. The φ_N_ angle is defined by extending the backbone (magenta line) to the center of the amino functional group. The inset shows the rule for classifying angles as cis (C), gauche (G), anticlinal (A), or trans (T).

The descriptive names are based on the torsion angles in the molecule, as defined in Figure 1. Although Ala_5_ has 20 rotable bonds, only 10 vary among the most stable conformers. The 5 {ω} angles are locked in the trans configuration by the π nature of the bond orbital, and the 5 {χ} angles prefer the trans (equivalently gauche) configuration because it minimizes steric repulsion in the side chain. The remaining 10 informative angles are classified as cis, gauche, anticlinal, or trans and then concatenated into a 10-letter string, N-terminus leftmost. If there are β-turns (H-bond between residues *i* and *i* + 3), γ-turns (*i* and *i*+2), or a C5 ring at the N-terminus, then the enclosed angles are replaced with the type of the turn. Because these descriptive names are quite long, we will use shortened names A*n*, meaning a conformer of Gibbs energy rank *n*.

We have also analyzed conformer geometries relative to each other. The MRSSAD, a measure of conformer distance, has been calculated for every pair. Figure 3 shows the conformers and the MRSSAD between them as a graph. Pairs with a low MRSSAD (shown as a thick edge) are related by a small transformation. As an example, A8 and A15 differ only by 120° in ψ_C_, meaning rotation of the carboxyl group, which enables an additional H-bond in the former.

**Figure 2:**
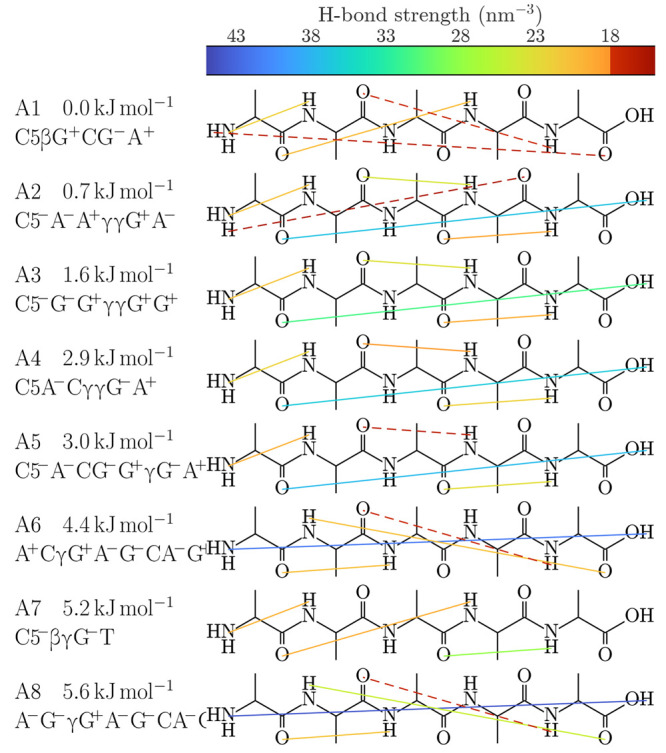
H-bonds present in the eight most stable conformers. The left side lists short names, Gibbs energies, and descriptive names. The colored lines represent H-bonds in the sense of eqs 2–4, with the strength indicated by the color. Intramolecular interactions with ρ in the range of 15–18 nm^–3^ are drawn as dashed lines. The descriptive names are constructed from the 10 torsion angles {φ} and {ψ} as defined in Figure 1 and turns caused by H-bonds.

The H-bonds visible in Figure 2 are based on our NCI analysis of the electron density. While its full results are available in the Supporting Information, we shall focus here on conformers A1–A8 and the H-bonds situated at their COOH and NH_2_ functional groups. These groups are of particular interest because their vibrational frequencies are sensitive to the type and strength of nearby H-bonds.

**Figure 3:**
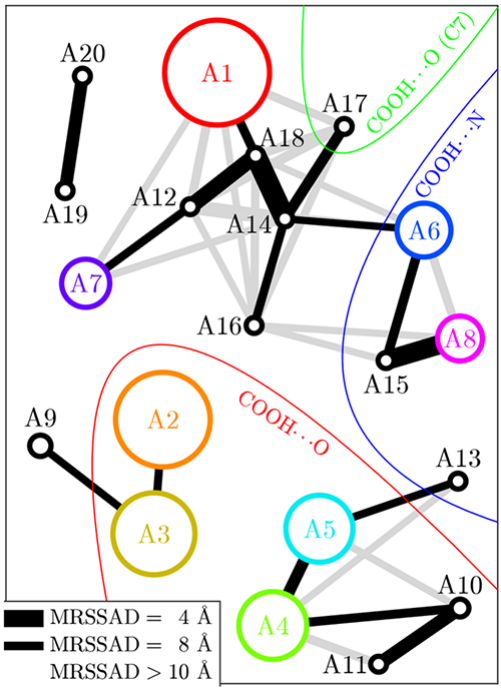
Twenty most abundant conformers visualized as a graph. Each node represents a conformer, and its area is proportional to the abundance. Edges are drawn between conformers with an MRSSAD of less than 10 Å, and thicker edges indicate a smaller MRSSAD. To increase visibility, the minimal spanning forest is colored black while the remaining edges are colored light gray. The colored regions tell where the COOH donates its H. Additionally, the eight most abundant conformers are highlighted with color.

Based on our NCI analysis, we find that the COOH group participates in up to two H-bonds, being able to donate with −H and accept with =O. The binding information is summarized by the colored regions in Figure 3. In A6 and A8, the COOH donates to the NH_2_ and accepts from the NH group furthest away (in topological distance), with the former bond being stronger. In A2–A5, the COOH donates to the =O group furthest away, although in A3 the bond is relatively weak. In A17, the COOH donates to the closest possible =O, forming a virtual ring of order 7. Finally, in all other conformers, notably A1 and A7, the COOH is free from H-bonds.

The NH_2_ group is less useful for discerning conformers. It accepts one bond in every low-energy conformer (A1–A15). In A6, A8, A13, and A15, the NH_2_ accepts from COOH and from the closest NH in every other low-energy conformer, forming a virtual five-membered ring (C5). The same ring was seen in the abundant conformers of dialanine.^25^

### 3.2 IRMPD–VUV Spectrum Assignment

**Figure 4:**
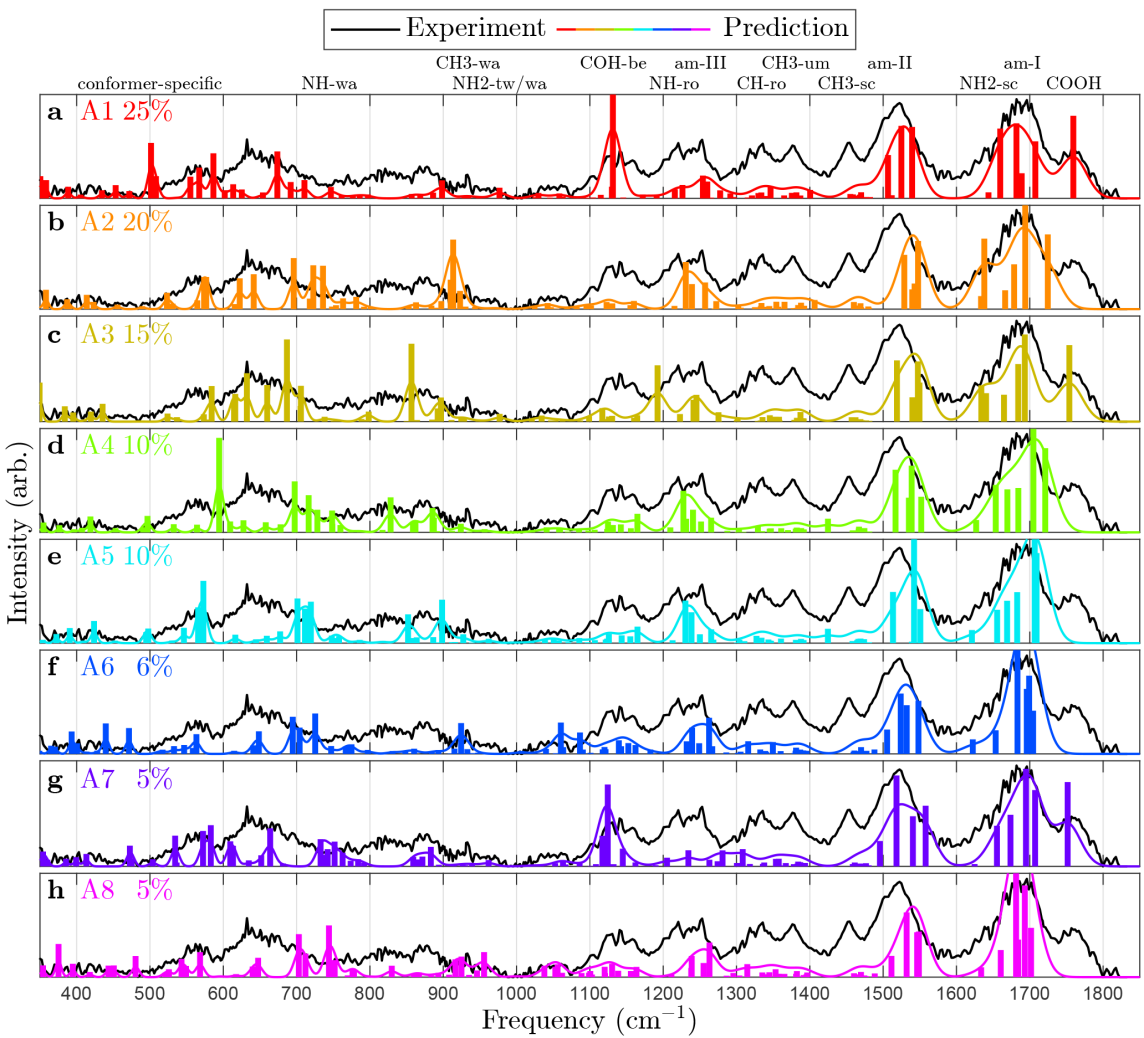
Experimental IRMPD–VUV spectrum of Ala_5_ (black lines) compared to predicted spectra (colored lines) of the eight most abundant conformers at 400 K, estimated from CBS-4 M Gibbs energies. The name and abundance of each conformer are listed (colored text). Abbreviations: wagging (wa), twisting (tw), bending (be), rocking (ro), amide (am), umbrella (um), and scissoring (sc).

Figure 4 shows the experimental IRMPD–VUV spectrum together with the calculated IR spectra of A1–A8. The experimental spectrum was measured in the range of 340–1820 cm^–1^ and plotted using eq 1. Comparing the experimental spectrum to the predicted vibrational modes allows for the qualitative inference of conformer populations. Of particular interest are the modes of the COOH functional group, whose frequencies depend strongly on the nearby H-bonds:

- The C=O stretching frequency is predicted to be 1760 cm^–1^ when the COOH group does not participate in H-bonds (A1 and A7) or only in a weak one (A3). In the presence of an H-bond, the frequency is red-shifted to about 1715 cm^–1^, very close to the amide I (carbonyl stretching) frequency. Based on the observed peak at 1760 cm^–1^, we conclude that at least one conformer A1, A3, or A7 must be present in our experiment.
- The COH bending frequency is predicted to be 1135 cm^–1^ in the absence of H-bonds (A1 and A7), 1190 cm^–1^ in the presence of a weak H-bond (A3), and 1220–1260 cm^–1^ otherwise. In the latter case, the frequency coincides with that of NH rocking modes, leading to a mixture of the modes. The experimental spectrum in the range of 1100–1280 cm^–1^ suggests that the population of A3 is small.
- The ω_C_ twisting frequency is predicted to be about 580 cm^–1^ in the absence of H-bonds (A1 and A7) and strongly blue-shifted otherwise. The blue shift increases with the H-bond strength. When the COOH donates to the NH_2_ group (A6 and A8), the H-bond is particularly strong and the frequency becomes 1060 cm^–1^. Otherwise, the COOH donates to a =O and the frequency lies in the range of 855–910 cm^–1^. Because of the wide range of predicted frequencies, it is difficult to assign this mode to any observed feature.

The conclusion to be drawn from these observations is that at least A1 or A7 is significantly populated. Their distinguishing property is that they do not form a loop from the COOH group to the first residue. Instead, their N-terminus and first peptide link prefer to form a C5-ring and a β-turn, excluding the COOH from intramolecular interactions.

Some observed features cannot be explained solely from A1 and A7. The peak at 630 cm^–1^ requires the presence of A2, A3, A6, or A8. Out of these, A2 has the best overall agreement with experiment and also the lowest energy. The addition of A2 also improves agreement in terms of the relative intensity of 1130 vs 1230 cm^–1^ and 1690 vs 1760 cm^–1^.

Another yet unexplained feature is that at 820 cm^–1^ neither A1, A2, nor A7 predicts a band. Based on its diffuse nature and approximate position, we speculate that it corresponds to COOH twisting in at least one member of A2–A5. In these conformers, the COOH H-bonds to the furthermost =O. We have previously seen that modes involving H-bonds are anharmonic and experimentally diffuse.^46^

In summary, we are able to infer the presence of A1/A7 and A2. The observed spectrum is consistent with the calculated Gibbs energies. It is not possible to distinguish similar conformers (such as A4 and A5) because they have similar spectra.

### 3.3 Static Harmonic vs Dynamic Spectrum

**Figure 5:**
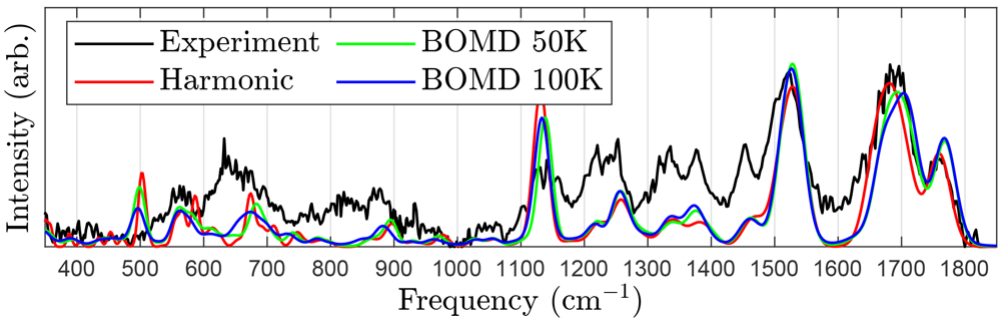
Experimental IRMPD–VUV spectrum of Ala_5_ (black) compared to the predicted spectra of A1 using harmonic analysis (red) and BOMD at 50 K (green) and 100 K (blue).

Figure 5 compares the B3LYP/Jun-cc-pVTZ harmonic predicted spectrum of A1 to that of B3LYP/N07D BOMD simulations. The predicted spectra differ below 1000 cm^–1^, but it is hard to appreciate which is better because of the congested experimental spectrum. Above 1000 cm^–1^, the three predicted spectra are quite similar. The most noticeable difference is that the dynamic spectra predict higher frequencies for the COOH modes.

The temperatures chosen (50 and 100 K in the BOMD simulations of Figure 5) are arguably too low to accurately describe anharmonic effects. For the width of the trajectory distribution to match the width of the nuclei probability density of a mode at ω = 1000 cm^–1^, the temperature must be around *T* = 1500 K . Sadly, running the BOMD at these temperatures produces an overly broad spectrum or even dissociates the molecule.

**Figure 6:**
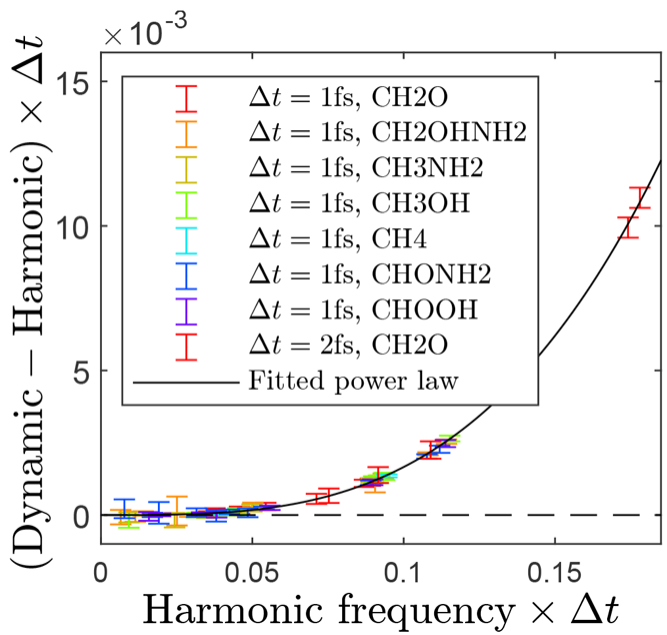
Difference between dynamic and harmonic dimensionless frequencies of small organic molecules (colored error bars). The error bar size is equal to the full width at half-maximum of the corresponding peak in the dynamic spectrum. The difference is well described by a power law (black line) with constant 2.95 and exponent 3.25.

We also investigated how the choice of time step influences the resulting spectrum. Figure 6 shows the frequencies of *T* = 1 K BOMD simulations of small organic molecules. In the limit *T*, Δ*t* → 0, the dynamic frequencies should approach the harmonic, and that is indeed seen. Moreover, the figure shows that each dynamic frequency ν_D_ is blue-shifted from the corresponding harmonic one ν_D_ by an amount well described by the power law
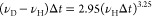
6This power law is seen to be independent of the molecule but still depends on the numerical integration scheme of the simulation. By applying it to our Ala_5_ simulations, which had a time step of Δ*t* = 0.5 fs and frequencies of interest of ν_H_ ≤ 1900 cm^–1^, we can estimate the frequency shift ν_D_–ν_H_ to be at most 2 cm^–1^ and thus negligible.

The existence of a molecule-independent relation such as eq 6 enables time savings in BOMD simulations. By running the simulation with a longer time step, one can obtain a blue-shifted spectrum. Then, by applying eq 6 in reverse (ν_D_ → ν_H_), the blue shift caused by the longer time step can be canceled out.

## 4 Conclusions

The IRMPD–VUV spectrum of Ala_5_ was obtained in the frequency range of 340–1820 cm^–1^. To our knowledge, this is the largest molecule to which this method has been applied. It is not conformer-selective; therefore, the spectrum obtained is a composition over many conformers.

BOMD simulations for spectrum prediction gave results similar to the harmonic analysis above 1000 cm^–1^. Below 1000 cm^–1^, there were differences, but without a conformer-specific spectrum, it is difficult to say which method is better. Considering that the BOMD simulations can be 1000 times more expensive, this makes an argument against the utility of BOMD in this context. Some runtime can be saved by increasing the time step using the blue-shift-compensating approach discussed earlier, but not enough to change the argument.

By comparing the observed spectrum with predictions at the B3LYP/Jun-cc-pVTZ level, we were able to infer the presence of two structure types. In the first, the COOH does not participate in H-bonds, and the peptide bond closest to the N-terminus forms both a C5-ring and a β-turn. In the second, the COOH is hydrogen bonded to the furthermost =O, and a γ-turn forms along the backbone. Gibbs energies at the CBS-4 M level support that these structures are the most populated.

The most abundant structure is stabilized by a C10 β-turn from residues 1 to 4 and to a lesser extent from residues 2 to 5. This can be interpreted as a very short 3_10_-helix, in which β-turns from residues *i* to *i* + 3 stabilize the structure. The same pattern of two adjacent β-turns exists in gas-phase Ac-Ala-Phe-Ala-NH_2_.^22,23^ Neutral polyalanine peptides of sufficient length are believed to form α-helices in the gas phase,^17^ but this has not been experimentally confirmed. Only by adding a spectroscopy-enabling component (H^+^, Na^+^, or a chromophore) has experiment been possible, and such components influence the tendency to form helices.^17^ Now, IRMPD–VUV spectroscopy offers the possibility of settling the question of helicity.

## Supporting Information Available

Conformers referred to in this article (PDF)
