# Supplementary material for: Indication of 310-Helix Structure in Gas-Phase Neutral Pentaalanine
Source: J Phys Chem A. 2023 Jan 20;127(4):938–45. doi: 10.1021/acs.jpca.2c07863 (PMC9900583; doi:10.1021/acs.jpca.2c07863)

Indication of 3_{10}-Helix Structure in Gas-Phase Neutral Pentaalanine

Åke Andersson,[†] Vasyl Yatsyna,[†,‡,¶] Mathieu Linares,[§] Anouk Rijs,[*,‡,∥] and Vitali Zhaunerchyk[*,†]

†*Department of Physics, University of Gothenburg, 41296 Gothenburg, Sweden*

‡*FELIX Laboratory, Institute for Molecules and Materials, Radboud University, Toernooiveld 7, 6525 ED Nijmegen, The Netherlands*

¶*Laboratoire de Chimie Physique Moléculaire, École Polytechnique Fédérale de Lausanne, EPFL SB ISIC LCPM, Station 6, CH-1015 Lausanne, Switzerland*

§*Laboratory of Organic Electronics and Group of Scientific Visualization Department of Science and Technology (ITN), Linköping University, 601 74 Norrköping, Sweden*

∥*Division of BioAnalytical Chemistry, AIMMS Amsterdam Institute of Molecular and Life Sciences, Vrije Universiteit Amsterdam, De Boelelaan 1108, 1081 HV, Amsterdam, The Netherlands.*

E-mail: a.m.rijs@vu.nl; vitali.zhaunerchyk@physics.gu.se

1 Supporting Information for "Evidence of 3_{10}-Helix Structure in Gas-Phase Neutral Pentaalanine"

The figures on page S1–20 show rotable backbone angles, 3D structure, and the strongest intramolecular interactions. Interaction strength is measured by bond distance Δr (Å) and electron density ρ (nm^{-3}).

The table below shows electronic and Gibbs energies along with Boltzmann populations. Structures were optimized using the B3LYP functional with added GD3BJ empirical dispersion on the Jun-cc-pVTZ basis set. Single-point energies were calculated at the the CBS-4M/Jun-cc-pVTZ level.

Table S1: Relative electronic and Gibbs energies at 400 K of the most stable conformers in kJ mol^{-1}, calculated with CBS-4M/Jun-cc-pVTZ. The two rightmost columns list corresponding Boltzmann populations and cumulative populations.

Conf.	E	G_{400}	p_{400}	$\sum p_{400}$
A1	17.9453	0.0000	0.246	0.246
A2	0.0000	0.6853	0.200	0.446
A3	3.3291	1.6331	0.150	0.596
A4	19.6020	2.9012	0.103	0.699
A5	16.4750	2.9773	0.100	0.799
A6	7.6638	4.4292	0.065	0.864
A7	20.7520	5.1880	0.052	0.916
A8	2.5074	5.6422	0.045	0.961
A9	27.1766	10.6018	0.010	0.971
A10	30.4112	11.7964	0.007	0.978
A11	32.5404	13.0802	0.005	0.983
A12	34.2733	14.2433	0.003	0.987
A13	19.3079	14.4192	0.003	0.990
A14	21.5186	14.9365	0.003	0.992
A15	27.5310	16.0156	0.002	0.994
A16	25.8323	18.1238	0.001	0.996
A17	18.1238	18.3102	0.001	0.997
A18	30.4400	18.5019	0.001	0.997
A19	26.0660	19.6256	0.001	0.998
A20	37.2821	20.2557	0.001	0.999

Figure S1: Geometry and H-bonds in conformer A1.

Conformer A1	φ_N	ψ_1	φ_2	ψ_2	φ_3	ψ_3	φ_4	ψ_4	φ_5	ψ_C
C5βG$^+$CG$^-$A$^+$	-151	-15	-63	118	54	31	67	13	-67	149

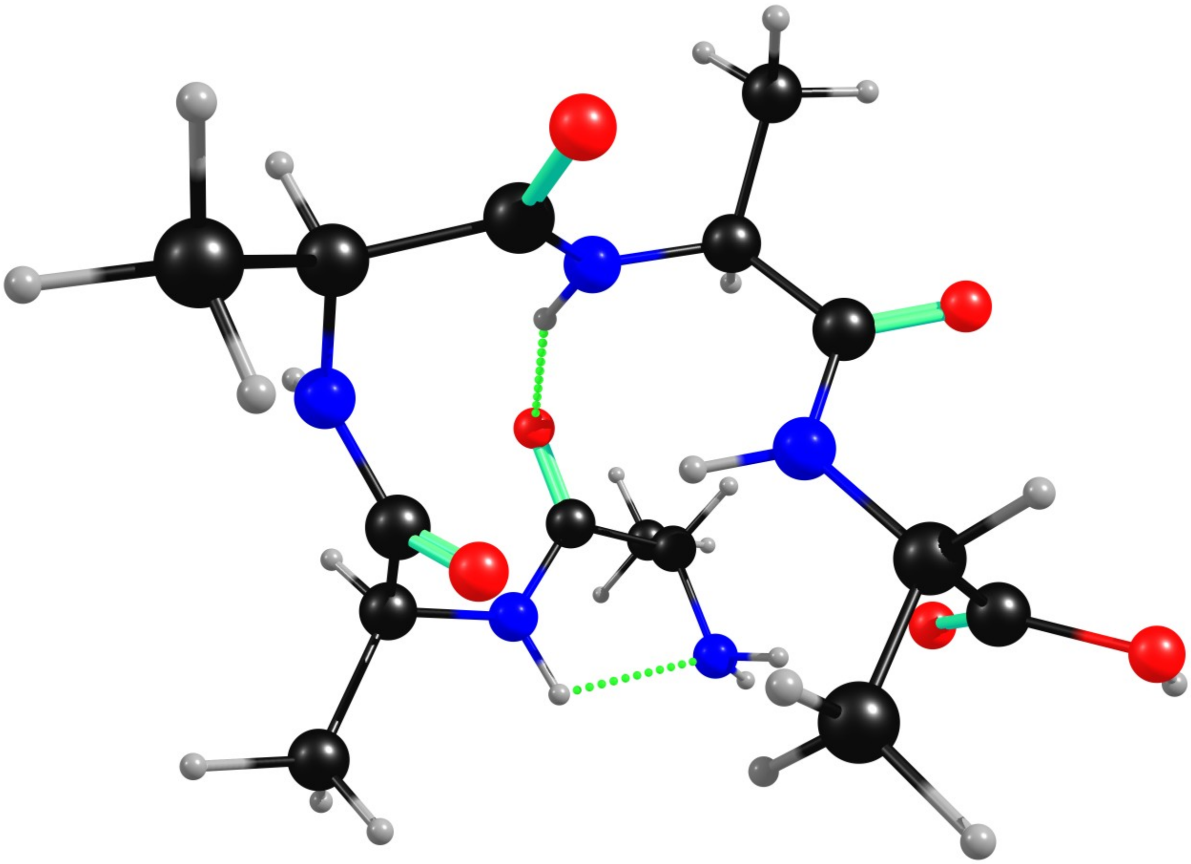

Strong interactions

Type	Δr	ρ
NH···N	2.1	21.9
NH···O	1.9	20.3
NH···O	2.0	17.6
NH···O	2.0	16.5

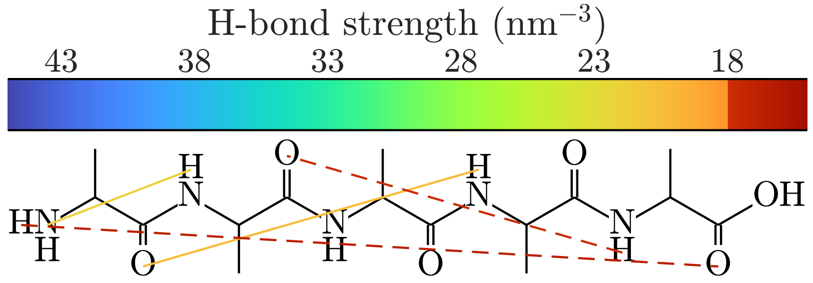

Figure S2: Geometry and H-bonds in conformer A2.

Conformer A2	φ_N	ψ_1	φ_2	ψ_2	φ_3	ψ_3	φ_4	ψ_4	φ_5	ψ_C
$C5^{-}A^{-}A^{+}\gamma\gamma G^{+}A^{-}$	-141	-18	-126	128	73	-60	-75	82	54	-141

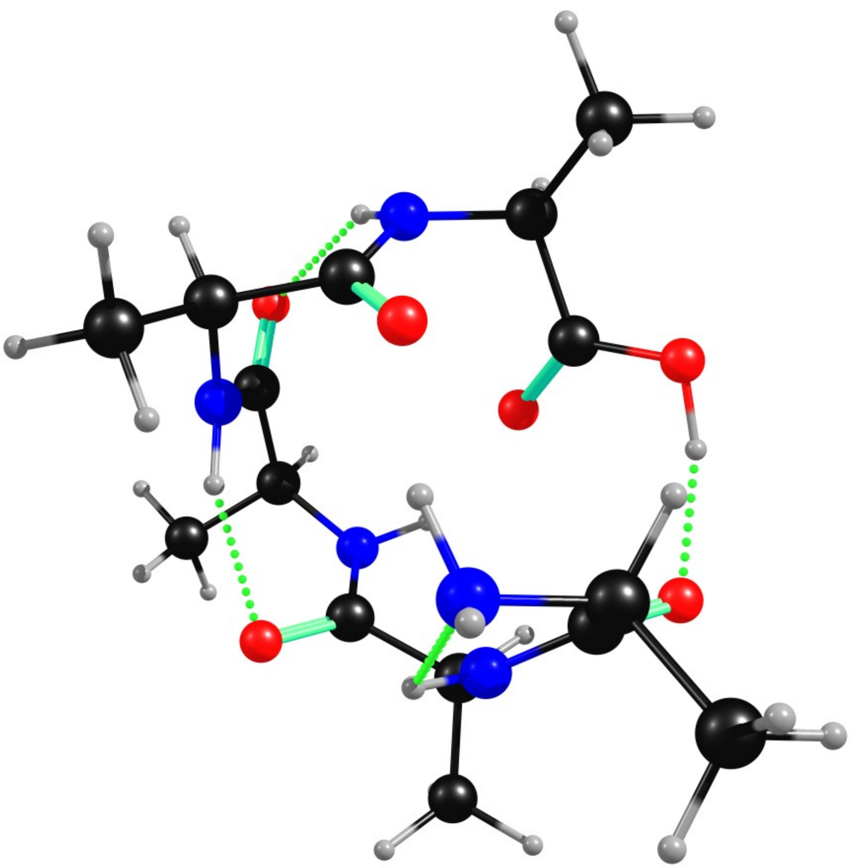

Strong interactions

Type	Δr	ρ
OH···O	1.7	37.0
NH···O	1.9	23.8
NH···N	2.1	20.8
NH···O	2.0	18.9
NH···O	2.1	15.7

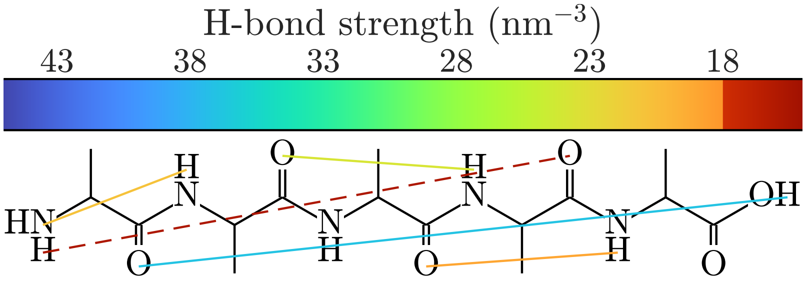

Figure S3: Geometry and H-bonds in conformer A3.

Conformer A3	φ_N	ψ_1	φ_2	ψ_2	φ_3	ψ_3	φ_4	ψ_4	φ_5	ψ_C
C5⁻G⁻G⁺γγG⁺G⁺	-144	-27	-82	89	72	-42	-74	85	51	39

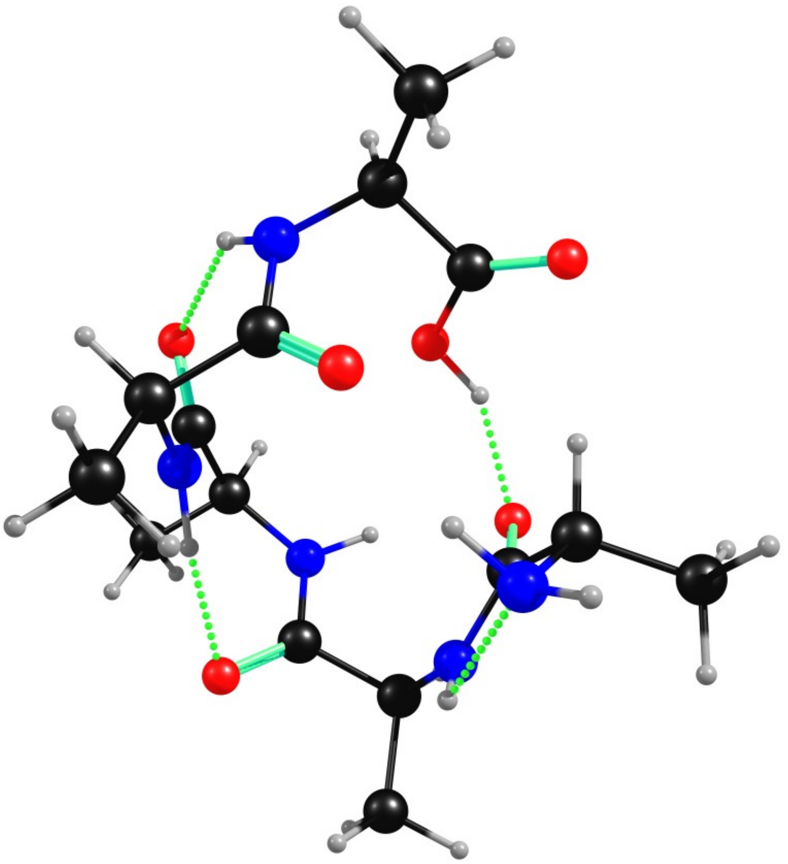

Strong interactions

Type	Δr	ρ
OH···O	1.8	31.4
NH···O	1.9	23.4
NH···N	2.1	20.9
NH···O	2.0	19.4

H-bond strength (nm^{-3})

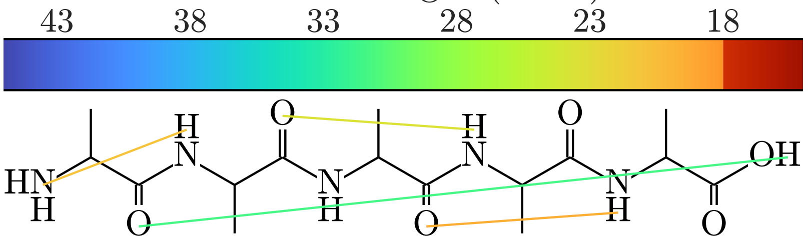

Figure S4: Geometry and H-bonds in conformer A4.

Conformer A4	φ_N	ψ_1	φ_2	ψ_2	φ_3	ψ_3	φ_4	ψ_4	φ_5	ψ_C
C5A$^-$C$\gamma\gamma$G$^-$A$^+$	155	11	-132	23	-84	71	69	-72	-74	139

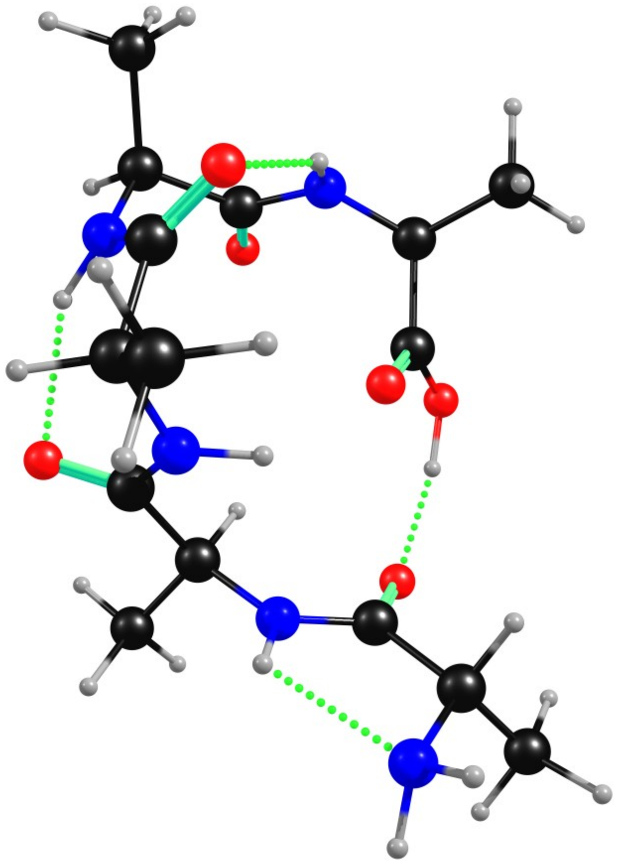

Strong interactions

Type	Δr	ρ
OH$\cdots$O	1.7	36.4
NH$\cdots$N	2.1	22.2
NH$\cdots$O	2.0	22.0
NH$\cdots$O	2.0	18.2

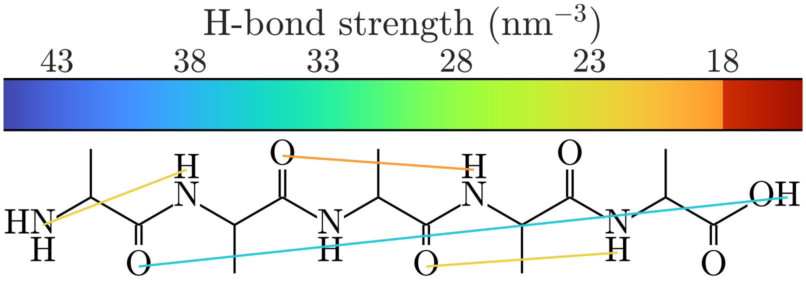

Figure S5: Geometry and H-bonds in conformer A5.

Conformer A5	φ_N	ψ_1	φ_2	ψ_2	φ_3	ψ_3	φ_4	ψ_4	φ_5	ψ_C
C5$^-$A$^-$CG$^-$G$^+\gamma$G$^-$A$^+$	-138	-24	-122	11	-87	69	69	-70	-74	134

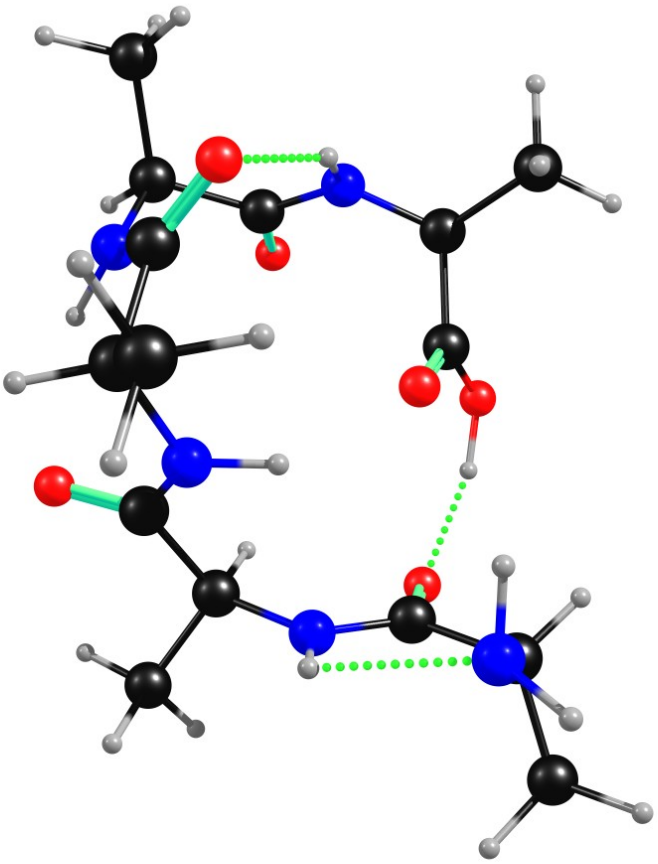

Strong interactions

Type	Δr	ρ
OH$\cdots$O	1.7	37.6
NH$\cdots$O	1.9	23.1
NH$\cdots$N	2.2	19.4
NH$\cdots$O	2.1	16.7

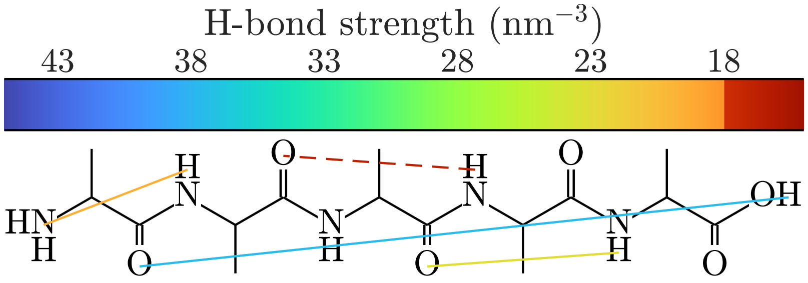

Figure S6: Geometry and H-bonds in conformer A6.

Conformer A6	φ_N	ψ_1	φ_2	ψ_2	φ_3	ψ_3	φ_4	ψ_4	φ_5	ψ_C
$A^{+}C\gamma G^{+}A^{-}G^{-}CA^{-}G^{+}$	125	-1	-79	82	55	-129	-75	-8	-101	59

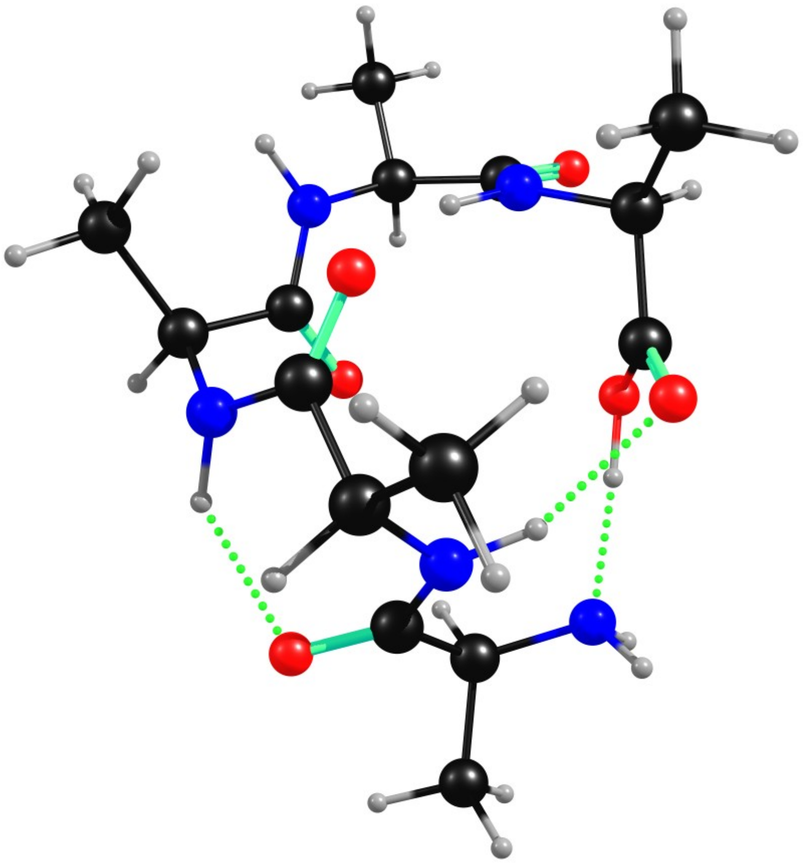

Strong interactions

Type	Δr	ρ
OH···N	1.7	41.8
NH···O	2.0	20.8
NH···O	2.0	20.6
NH···O	2.0	17.5

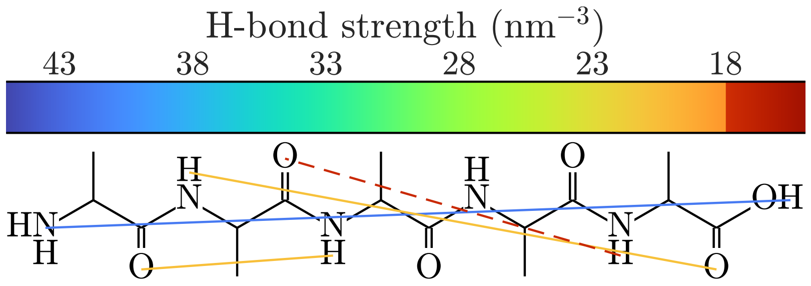

Figure S7: Geometry and H-bonds in conformer A7.

Conformer A7	φ_N	ψ_1	φ_2	ψ_2	φ_3	ψ_3	φ_4	ψ_4	φ_5	ψ_C
C5⁻$\beta\gamma$G⁻T	-137	-17	-64	-24	-122	36	69	-66	-63	166

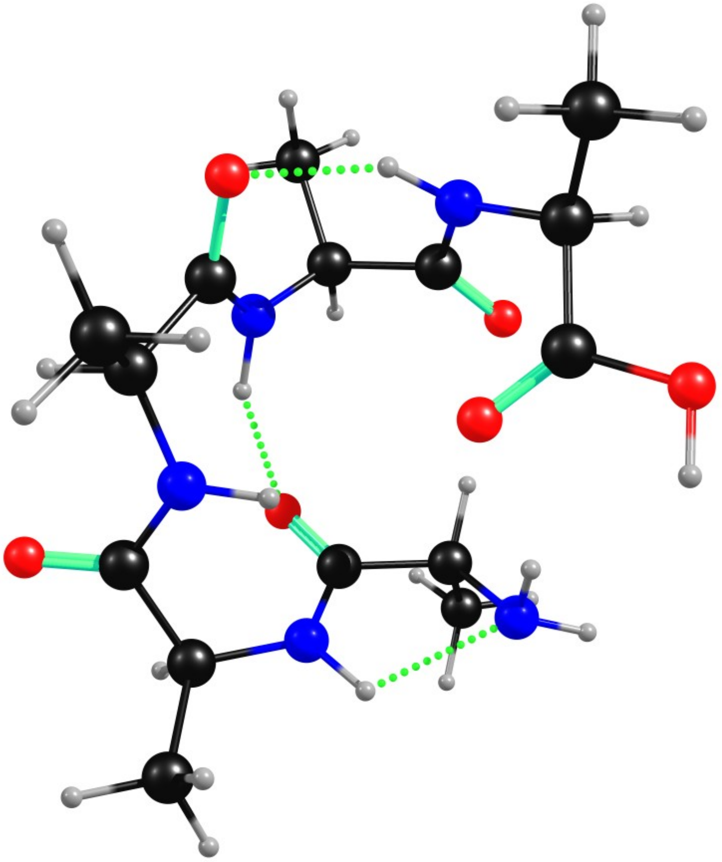

Strong interactions

Type	Δr	ρ
NH···O	1.8	28.0
NH···N	2.2	19.6
NH···O	2.0	19.4

H-bond strength (nm^{-3})

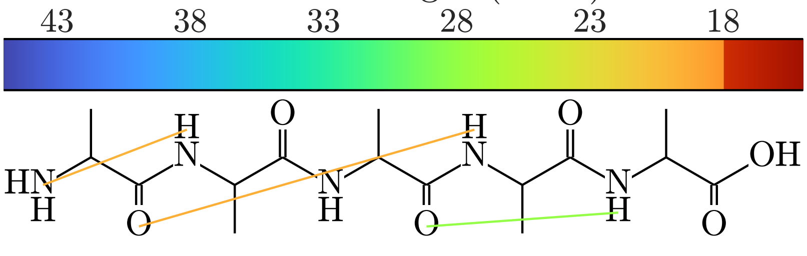

Figure S8: Geometry and H-bonds in conformer A8.

Conformer A8	φ_N	ψ_1	φ_2	ψ_2	φ_3	ψ_3	φ_4	ψ_4	φ_5	ψ_C
$A^-G^-\gamma G^+A^-G^-CA^-G^+$	-103	-71	-78	79	54	-126	-76	-8	-97	62

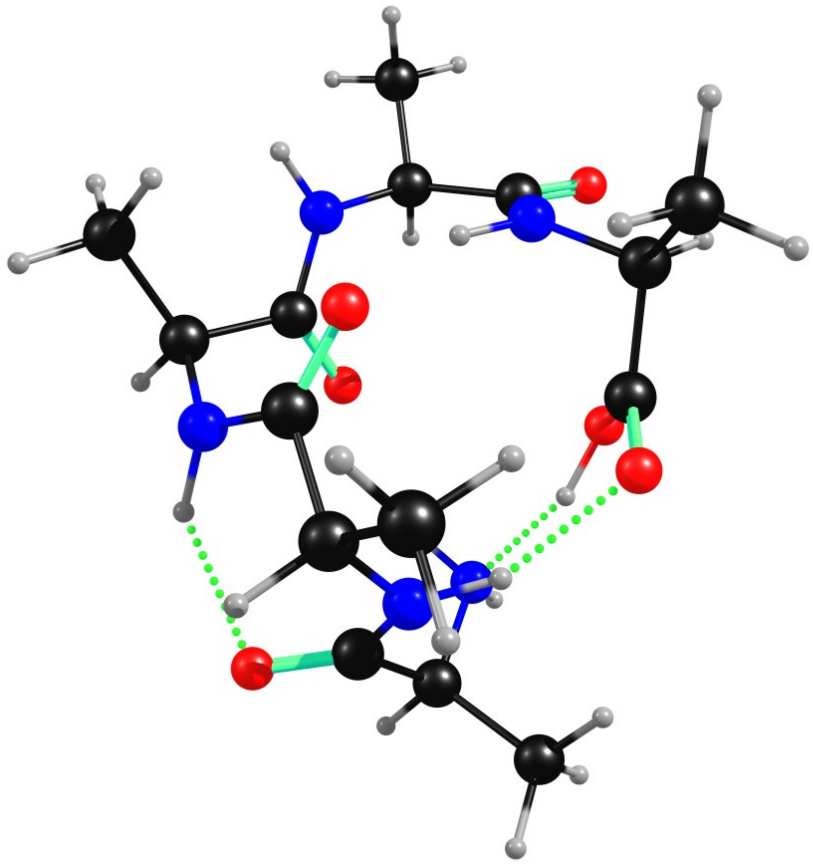

Strong interactions

Type	Δr	ρ
OH···N	1.7	43.9
NH···O	1.9	24.9
NH···O	2.0	21.0
NH···O	2.0	17.3

H-bond strength (nm^{-3})

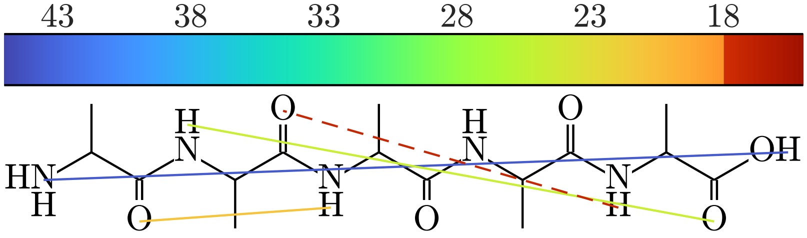

Figure S9: Geometry and H-bonds in conformer A9.

Conformer A9	φ_N	ψ_1	φ_2	ψ_2	φ_3	ψ_3	φ_4	ψ_4	φ_5	ψ_C
C5⁻γγγA⁻C	-142	-22	-79	84	73	-52	-77	77	-109	-18

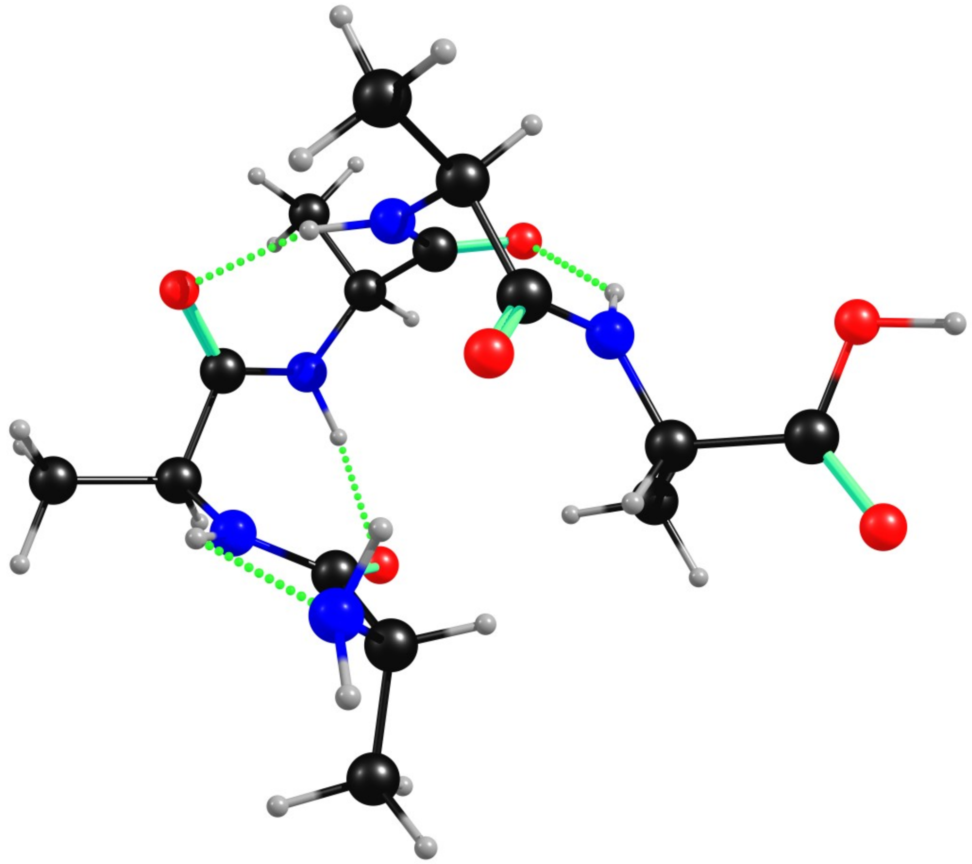

Strong interactions

Type	Δr	ρ
NH···O	1.9	24.6
NH···N	2.1	21.0
NH···O	2.0	20.5
NH···O	2.1	18.7
NH···O	2.0	15.9

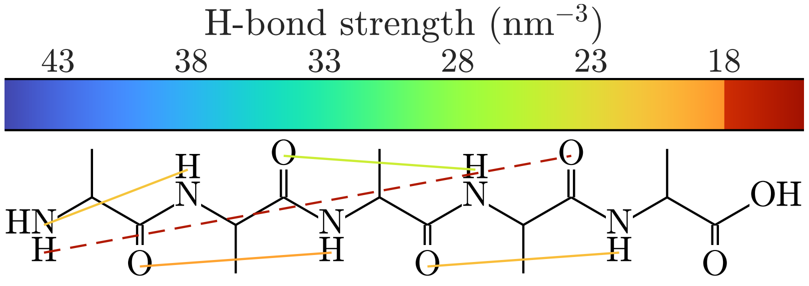

Figure S10: Geometry and H-bonds in conformer A10.

Conformer A10	φ_N	ψ_1	φ_2	ψ_2	φ_3	ψ_3	φ_4	ψ_4	φ_5	ψ_C
C5⁻G⁻G⁺γγG⁻C	-147	-16	-85	49	-80	71	69	-73	-80	-22

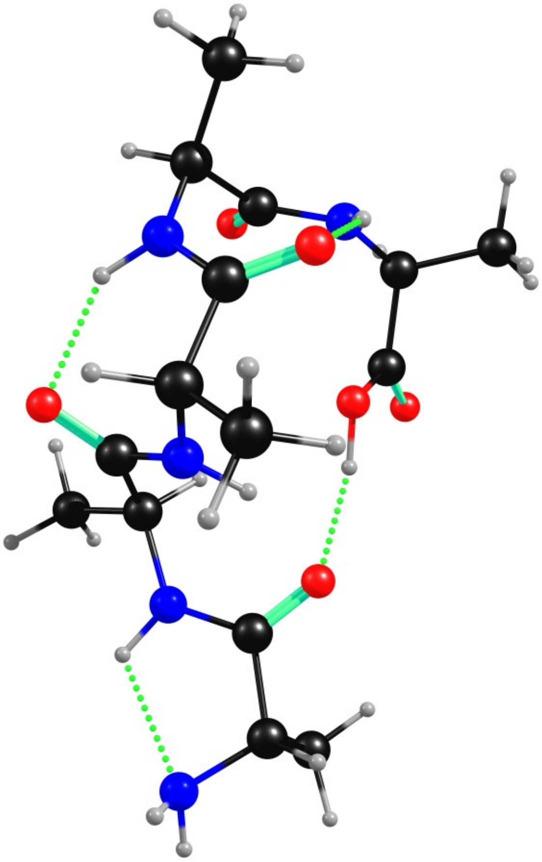

Strong interactions

Type	Δr	ρ
OH···O	1.8	28.2
NH···O	2.0	20.6
NH···O	2.0	20.5
NH···N	2.1	20.4
NH···O	2.1	16.6

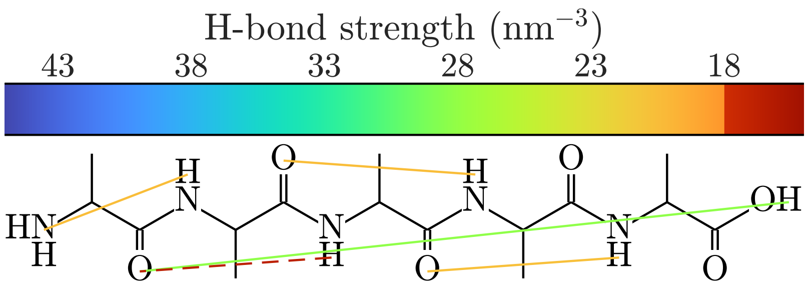

Figure S11: Geometry and H-bonds in conformer A11.

Conformer A11	φ_N	ψ_1	φ_2	ψ_2	φ_3	ψ_3	φ_4	ψ_4	φ_5	ψ_C
C5G⁻G⁺γγG⁻C	155	7	-84	49	-80	71	69	-73	-80	-22

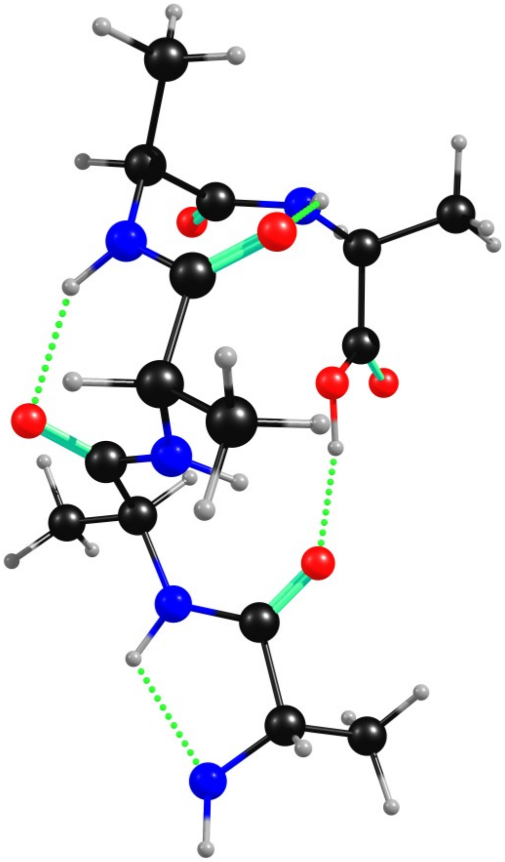

Strong interactions

Type	Δr	ρ
OH···O	1.8	28.3
NH···N	2.1	21.7
NH···O	2.0	20.7
NH···O	2.0	20.3
NH···O	2.1	16.7

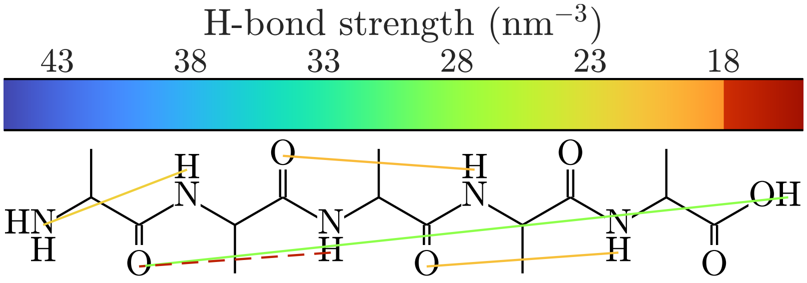

Figure S12: Geometry and H-bonds in conformer A12.

Conformer A12	φ_N	ψ_1	φ_2	ψ_2	φ_3	ψ_3	φ_4	ψ_4	φ_5	ψ_C
C5⁻$\beta\beta$A⁻G⁺	-137	-19	-66	111	54	33	62	21	-138	37

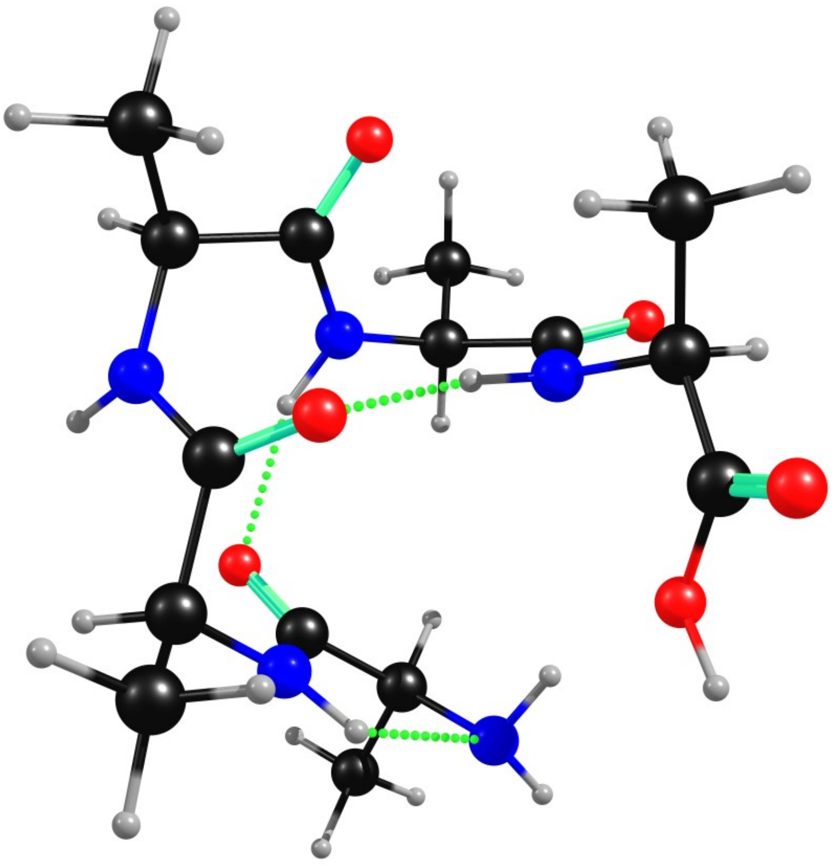

Strong interactions

Type	Δr	ρ
NH···N	2.2	19.8
NH···O	2.0	19.4
NH···O	2.0	18.7
44···O	2.3	15.7
NH···N	2.3	15.2

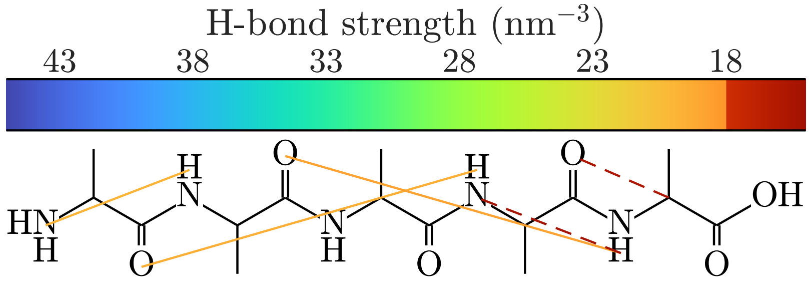

Figure S13: Geometry and H-bonds in conformer A13.

Conformer A13	φ_N	ψ_1	φ_2	ψ_2	φ_3	ψ_3	φ_4	ψ_4	φ_5	ψ_C
$A^+A^+\gamma\gamma\gamma A^-G^-$	109	123	73	-43	-78	83	73	-52	-131	-66

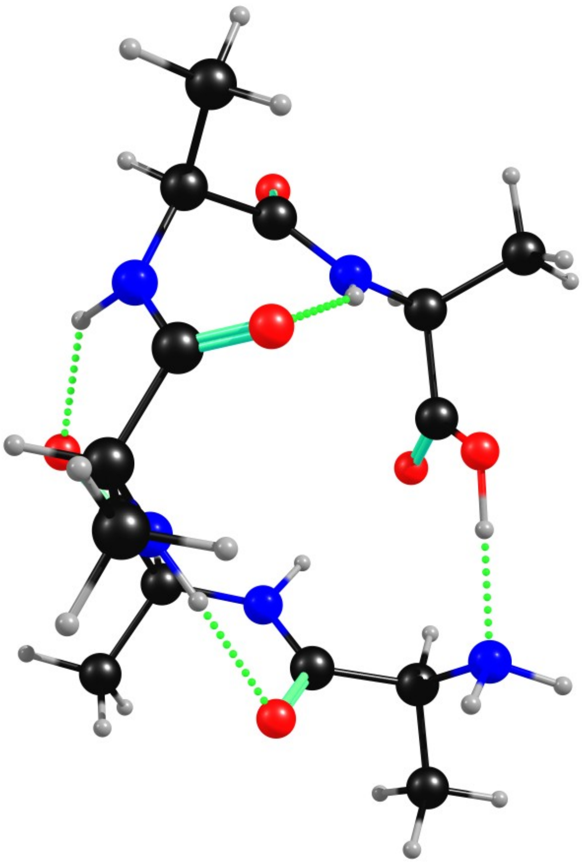

Strong interactions

Type	Δr	ρ
OH···N	1.7	44.7
NH···O	1.9	24.8
NH···O	2.0	21.5
NH···O	2.0	19.9

H-bond strength (nm^{-3})

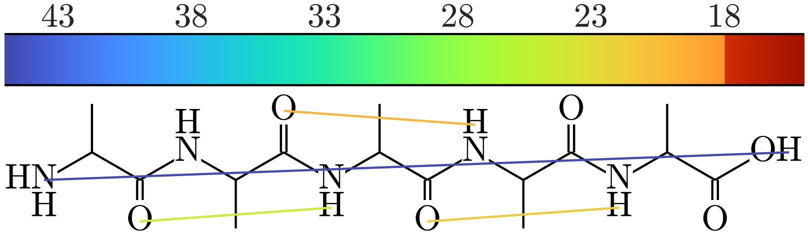

Figure S14: Geometry and H-bonds in conformer A14.

Conformer A14	φ_N	ψ_1	φ_2	ψ_2	φ_3	ψ_3	φ_4	ψ_4	φ_5	ψ_C
C5$^-\gamma$G$^+$G$^+\gamma$G$^-$T	-144	-15	-75	88	52	48	72	-46	-55	150

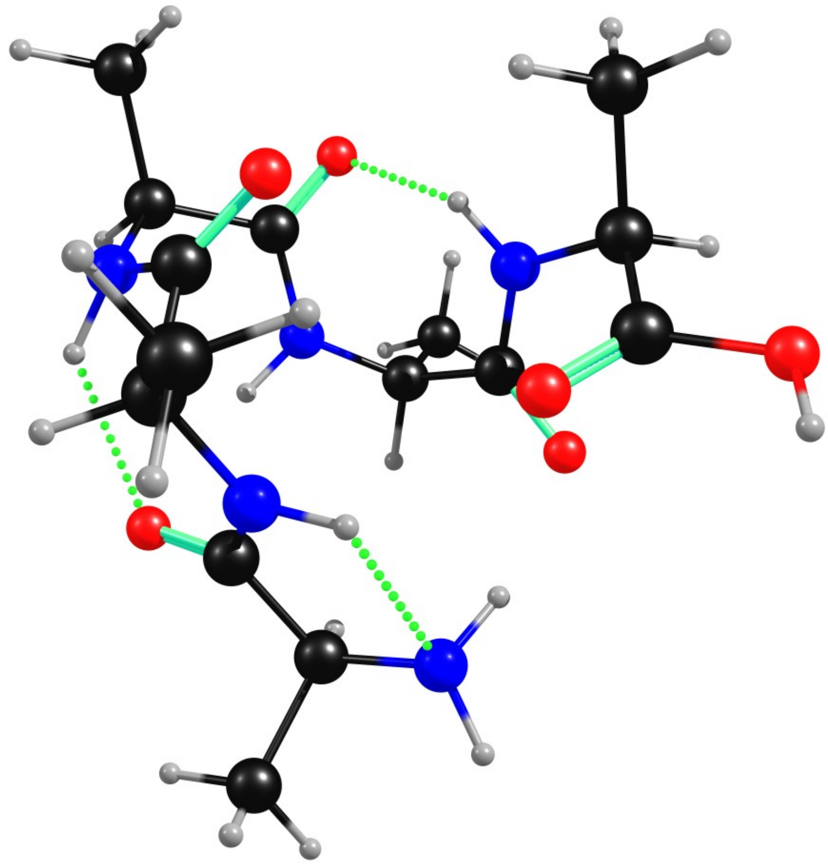

Strong interactions

Type	Δr	ρ
NH$\cdots$O	2.0	20.9
NH$\cdots$N	2.2	18.9
NH$\cdots$O	2.1	18.2
NH$\cdots$O	2.1	15.6

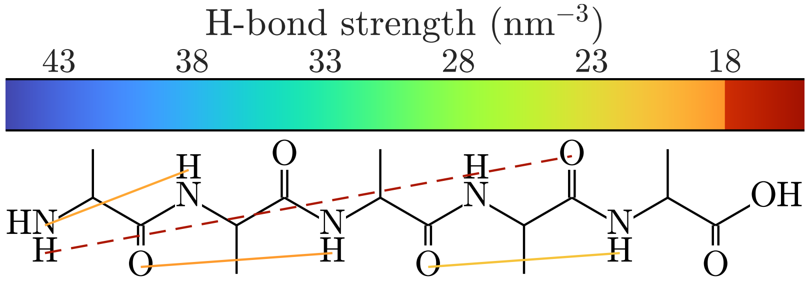

Figure S15: Geometry and H-bonds in conformer A15.

Conformer A15	φ_N	ψ_1	φ_2	ψ_2	φ_3	ψ_3	φ_4	ψ_4	φ_5	ψ_C
A⁻G⁻γG⁺A⁻G⁻CG⁻G⁻	-117	-55	-78	77	53	-127	-79	-7	-81	-57

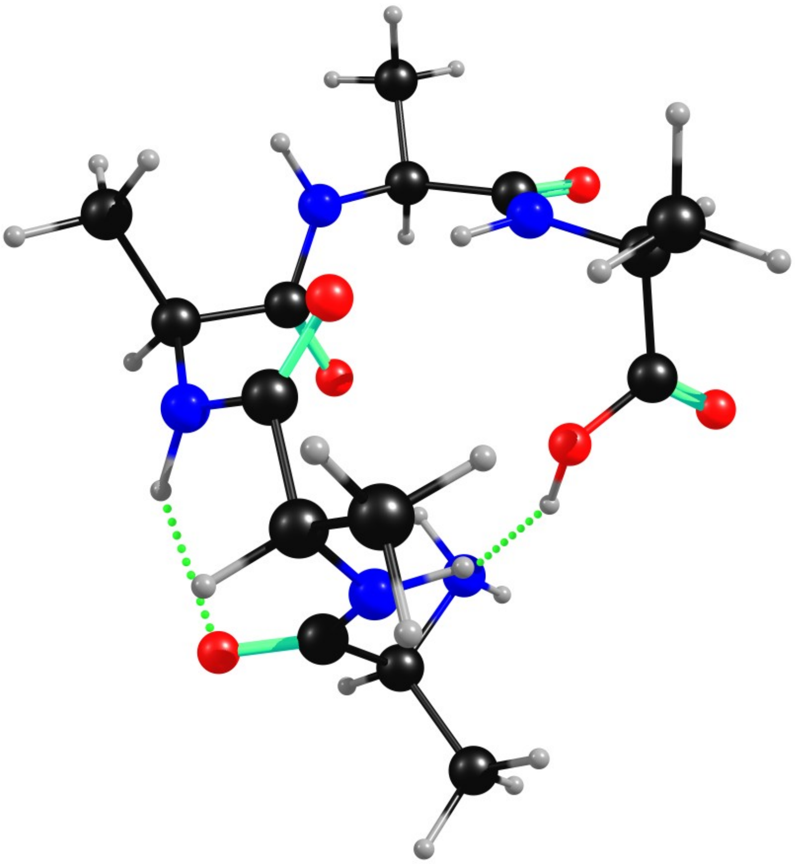

Strong interactions

Type	Δr	ρ
OH···N	1.7	42.2
NH···O	2.0	19.5
NH···O	2.0	16.0

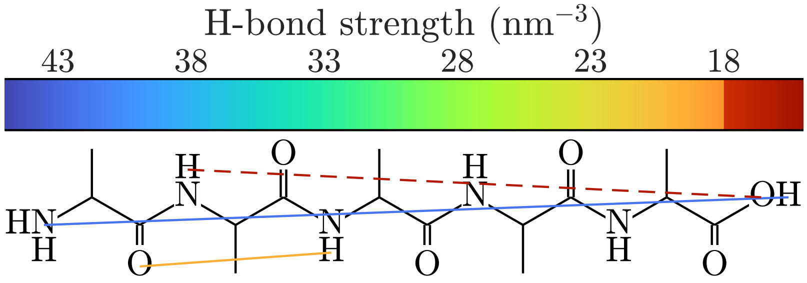

Figure S16: Geometry and H-bonds in conformer A16.

Conformer A16	φ_N	ψ_1	φ_2	ψ_2	φ_3	ψ_3	φ_4	ψ_4	φ_5	ψ_C
$A^+G^-G^-G^+G^+G^+A^+CG^-T$	142	-68	-80	77	52	45	91	-28	-70	158

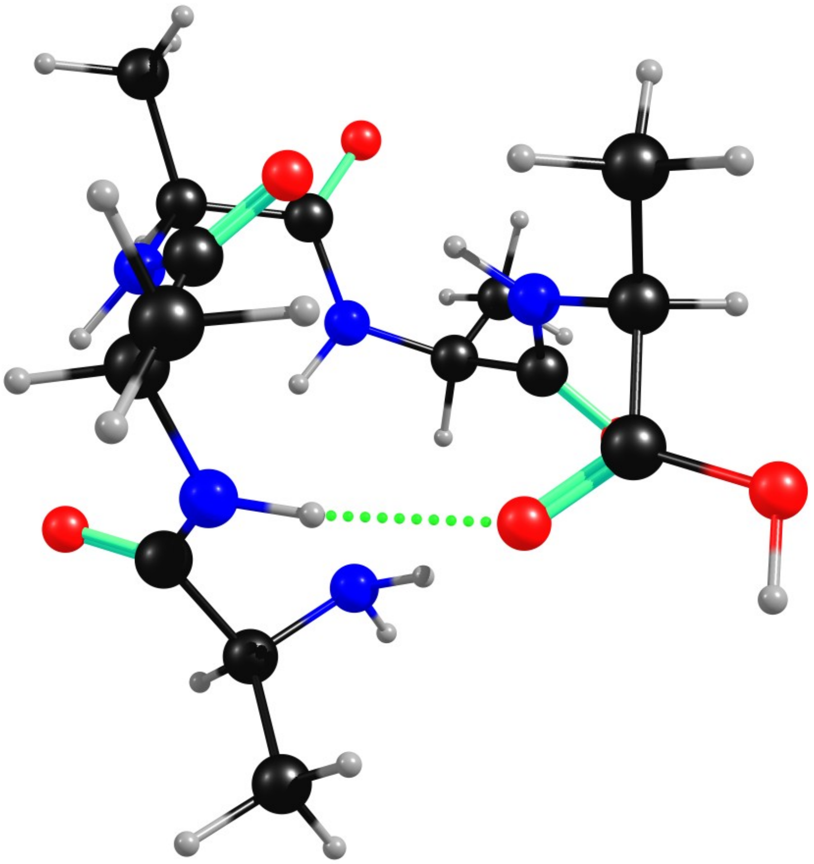

Strong interactions

Type	Δr	ρ
NH···O	2.0	18.5
NH···O	2.1	17.6
NH···O	2.0	16.6

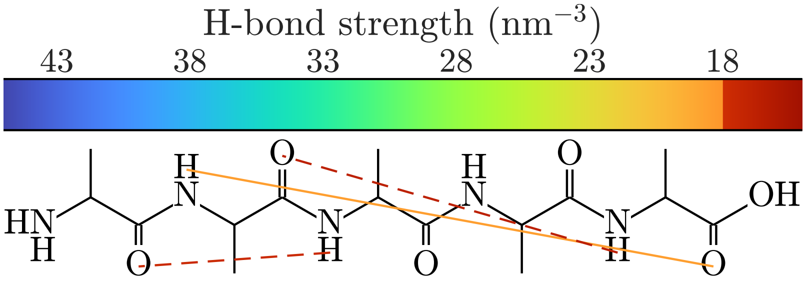

Figure S17: Geometry and H-bonds in conformer A17.

Conformer A17	φ_N	ψ_1	φ_2	ψ_2	φ_3	ψ_3	φ_4	ψ_4	φ_5	ψ_C
$C5^{-}G^{-}A^{+}G^{+}G^{+}\gamma G^{-}G^{+}$	-145	-18	-72	94	47	47	70	-55	-70	56

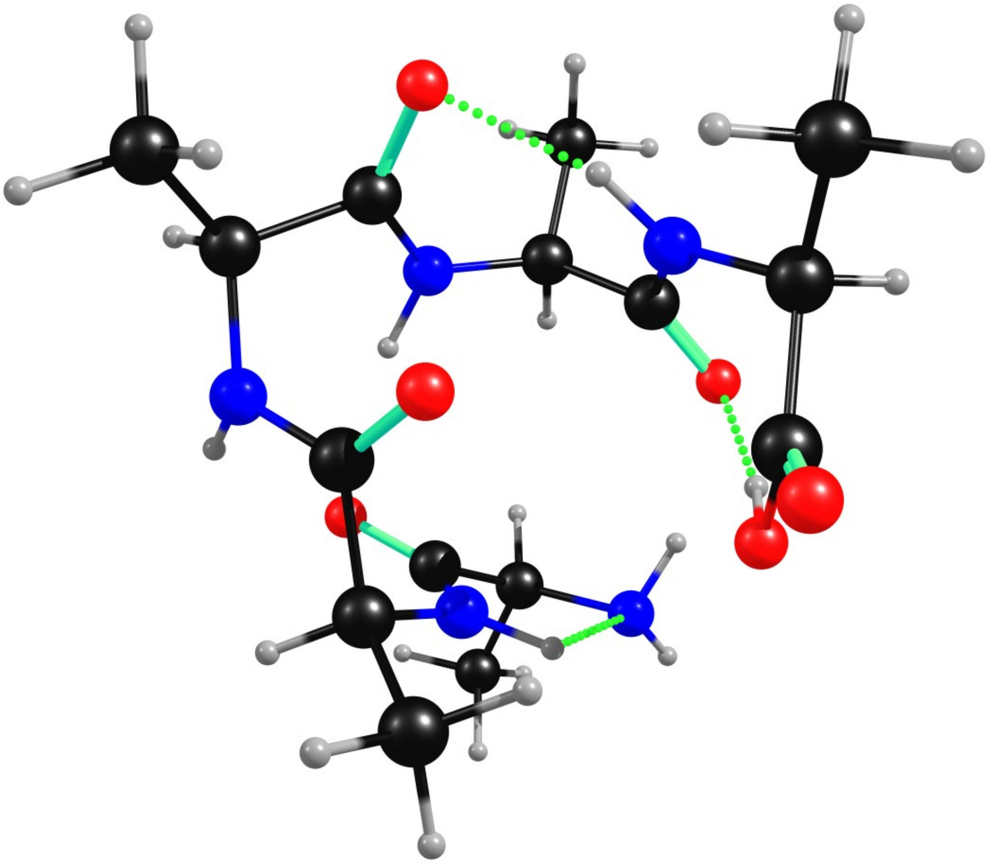

Strong interactions

Type	Δr	ρ
OH···O	1.7	37.2
NH···O	1.9	22.6
NH···N	2.2	18.8
NH···O	2.1	15.9
NH···O	2.1	15.4
NH···O	2.2	15.3

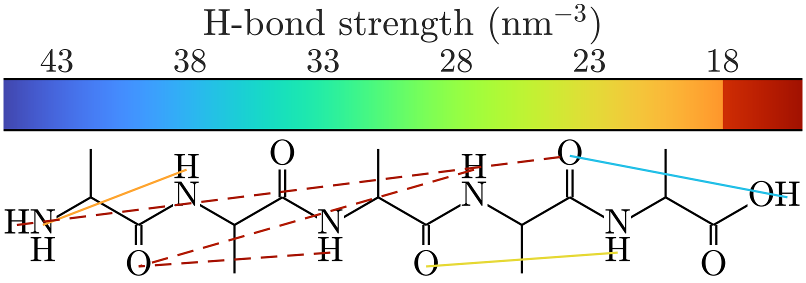

Figure S18: Geometry and H-bonds in conformer A18.

Conformer A18	φ_N	ψ_1	φ_2	ψ_2	φ_3	ψ_3	φ_4	ψ_4	φ_5	ψ_C
CCG⁻A⁺βA⁻T	13	-22	-76	97	52	41	68	20	-136	177

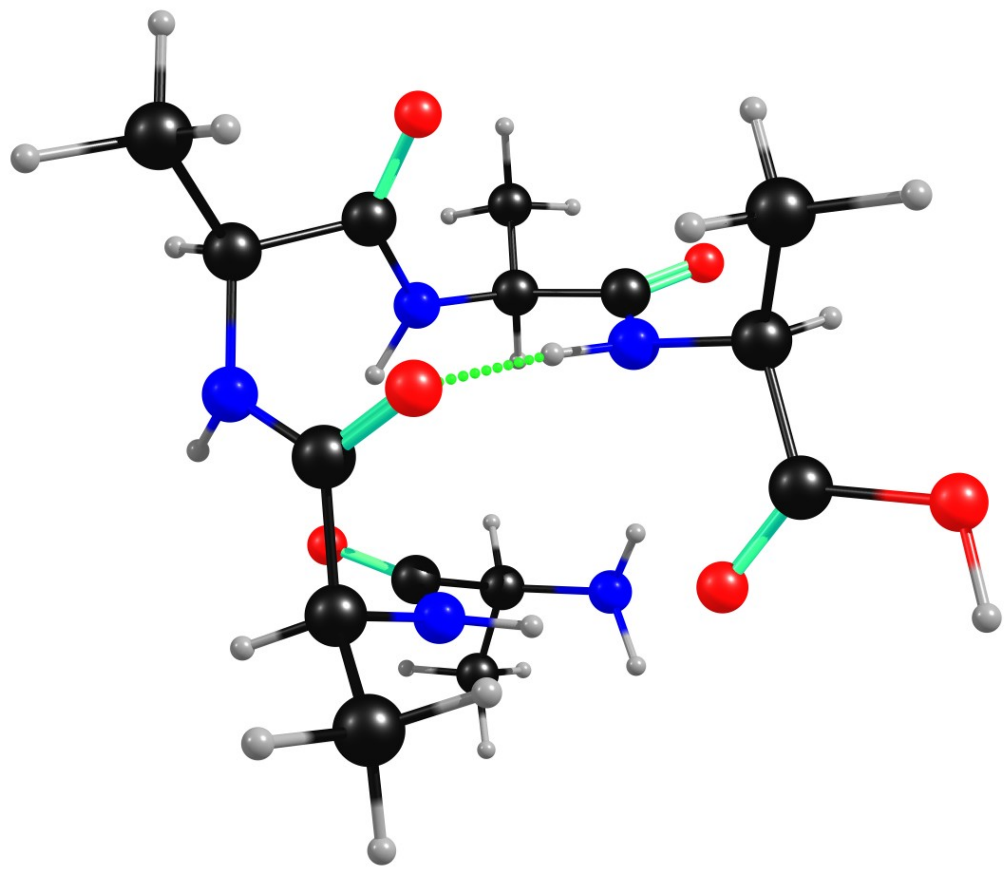

Strong interactions

Type	Δr	ρ
NH···O	2.0	18.0
NH···N	2.3	15.5
44···O	2.3	15.4
NH···O	2.1	15.1

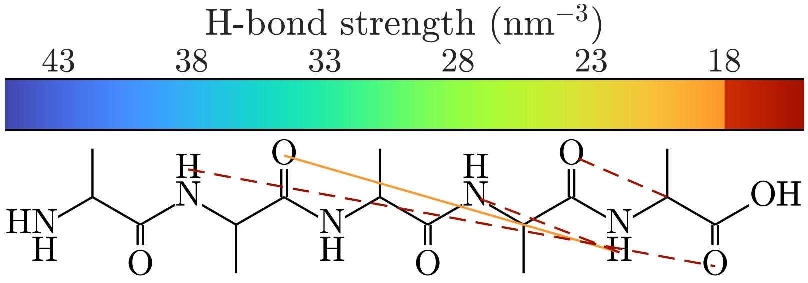

Figure S19: Geometry and H-bonds in conformer A19.

Conformer A19	φ_N	ψ_1	φ_2	ψ_2	φ_3	ψ_3	φ_4	ψ_4	φ_5	ψ_C
$TG^{+}\gamma\beta G^{+}A^{-}$	-151	75	70	-67	-58	-33	-116	42	54	-140

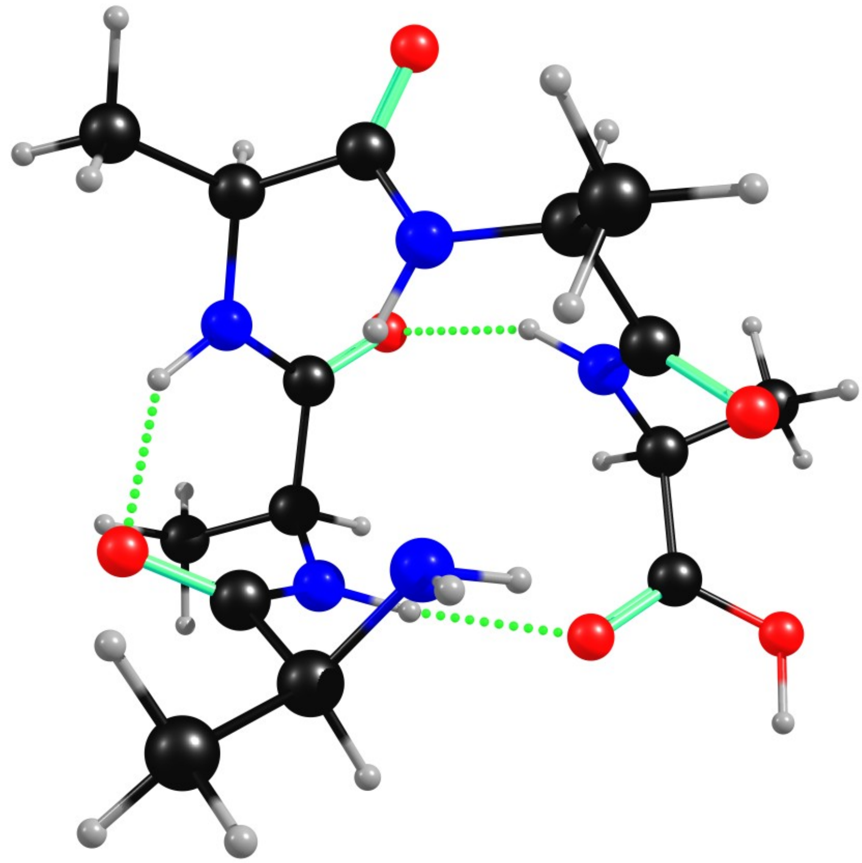

Strong interactions

Type	Δr	ρ
NH$\cdots$O	1.9	24.1
NH$\cdots$O	2.0	19.9
NH$\cdots$O	2.0	18.8
34$\cdots$O	2.3	15.2

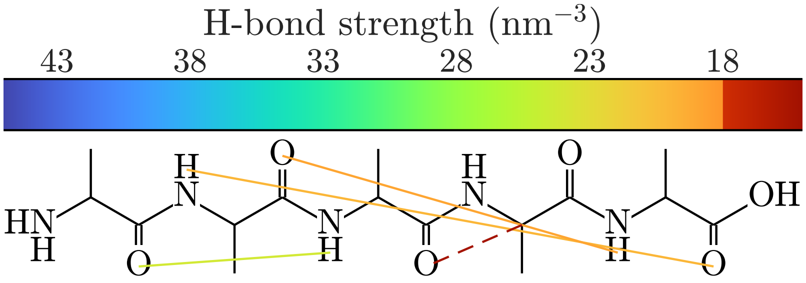

Figure S20: Geometry and H-bonds in conformer A20.

Conformer A20	φ_N	ψ_1	φ_2	ψ_2	φ_3	ψ_3	φ_4	ψ_4	φ_5	ψ_C
$C5^{+}\gamma G^{-}CA^{-}G^{+}G^{+}A^{-}$	143	10	75	-61	-62	-21	-112	40	58	-146

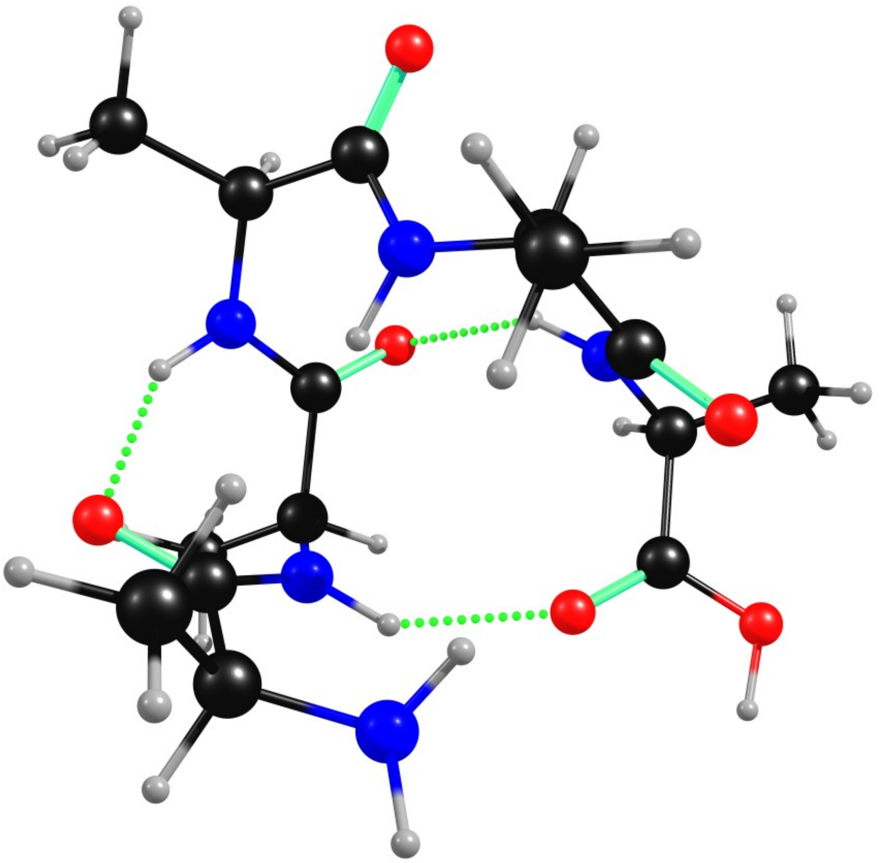

Strong interactions

Type	Δr	ρ
NH···O	1.9	26.8
NH···N	2.1	20.0
NH···O	2.0	17.6
NH···N	2.3	17.3

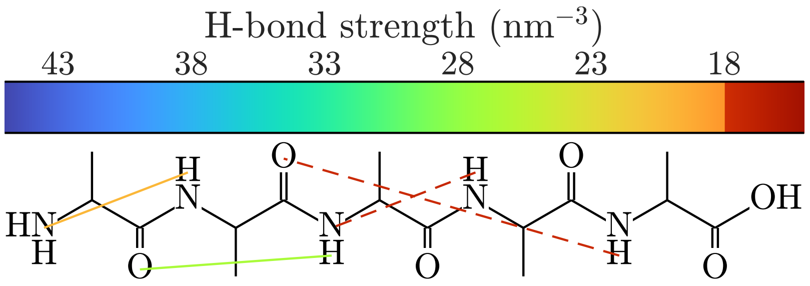

Supplement: Supplementary file 1 — jp2c07863_si_001.pdf [file jp2c07863_si_001.pdf]
